# QSAR-Guided In Silico Design of Piperazinylanthranilamide Factor Xa Inhibitors: Applicability-Domain Assessment, ADMET Profiling, Molecular Docking, Molecular Dynamics, and MM/GBSA

**DOI:** 10.3390/ijms27209204

**Published:** 2026-10-16

**Authors:** Paweł Gordon, Michał Janiak, Marcin Gackowski, Monika Zielińska-Pisklak, Łukasz Szeleszczuk

**Affiliations:** 1 University of Health Sciences in Bydgoszcz, Jagiellońska 4 Str., 85-067 Bydgoszcz, Poland; p.gordon@wsnoz.edu.pl; 2 Department of Toxicology and Bromatology, Faculty of Pharmacy, L. Rydygier Collegium Medicum in Bydgoszcz, Nicolaus Copernicus University in Toruń, A. Jurasza 2 Street, 85-089 Bydgoszcz, Poland; michal.janiak@doktorant.umk.pl (M.J.); marcin.gackowski@cm.umk.pl (M.G.); 3 Doctoral School of Medical and Health Sciences, Ludwik Rydygier Collegium Medicum, Nicolaus Copernicus University in Toruń, Jagiellońska 13-15 Str., 85-067 Bydgoszcz, Poland; 4 Department of Pharmaceutical Chemistry and Biomaterials, Faculty of Pharmacy, Medical University of Warsaw, 1 Banacha Str., 02-093 Warsaw, Poland; mpisklak@wum.edu.pl; 5 Department of Organic and Physical Chemistry, Medical University of Warsaw, 1 Banacha Str., 02-097 Warsaw, Poland

**Keywords:** factor Xa, piperazinylanthranilamides, QSAR, MARSplines, applicability domain, molecular docking, molecular dynamics, MM/GBSA, ADMET

## Abstract

An integrated computational workflow was developed to prioritize piperazinylanthranilamide factor Xa (FXa) inhibitor candidates. Twenty literature compounds were geometry-optimized and described using alvaDesc to construct a constrained additive multivariate adaptive regression splines model. Internal validation and an assessment of the applicability domain guided the evaluation of 20 prospective analogs. Ten selected analogs and two same-series references were docked to FXa, with apixaban as the crystallographic comparator. Thirteen initial 100 ns molecular dynamics simulations were supplemented by two independent runs for each of PAX-M09, PAX-M15, and PAX-M18. Binding energetics were assessed using molecular mechanics/generalized Born surface area (MM/GBSA), alongside in silico developability profiling. The model achieved R^2^ = 0.904 and an internal leave-one-out Q^2^ of 0.846. PAX-M09 was the strongest strict in-domain prediction, whereas PAX-M15 was borderline. Apixaban redocking yielded a root-mean-square deviation (RMSD) of 0.953 Å. Although PAX-M17 and PAX-M19 ranked highest in docking, subsequent analyses prioritized PAX-M15, PAX-M09, and PAX-M18. PAX-M15 showed the most consistent positional behavior across replicates and consistently favorable run-specific MM/GBSA estimates (−68.76, −70.11, and −69.42 kcal/mol). PAX-M09 and PAX-M18 showed greater between-run heterogeneity. Solubility, metabolic, and toxicity liabilities remained important. The results support experimental investigation of the three candidates without changing their applicability-domain classifications or establishing superior experimental potency.

## 1. Introduction

Thromboembolic disease remains a major cause of morbidity and mortality, and anticoagulant therapy is central to the prevention and treatment of venous and arterial thrombotic events [1]. Factor Xa (FXa) occupies a key amplification point in the coagulation cascade, where it catalyzes prothrombin activation and therefore regulates downstream thrombin generation. Direct inhibition of FXa has become an established anticoagulant strategy, although bleeding, renal impairment, drug–drug interactions, peri-procedural management, and reversal of anticoagulation remain clinically relevant challenges [2,3]. These considerations continue to motivate the search for structurally differentiated FXa inhibitors and improved understanding of the molecular features that govern activity.

Direct small-molecule FXa inhibitors generally exploit a non-linear ligand architecture in which hydrophobic P1 and P4 regions occupy the S1 and S4 recognition pockets, respectively. Anthranilamide-derived scaffolds have been used repeatedly to organize these recognition elements, and previous medicinal chemistry studies have demonstrated that systematic changes at the P1, central scaffold, and P4 regions can strongly modulate potency, selectivity, anticoagulant activity, and pharmacokinetic behavior [4,5,6,7]. The piperazinylanthranilamide series reported by Khadse et al. is particularly attractive for descriptor-based modeling because it combines a common reverse-anthranilamide framework with controlled variation in three chemically interpretable regions [4].

In the source study, 59 piperazinylanthranilamides were first screened at 20 µM, and the 20 compounds producing more than 60% FXa inhibition were subsequently subjected to IC_50_ determination [4]. These twenty derivatives span IC_50_ values from 0.61 to 39.9 µM. Compounds **6y** and **7f** were the most potent members of the series, with IC_50_ values of 0.61 and 0.74 µM, respectively, while 7v provided an additional high-activity example with an IC_50_ of 1.8 µM. The source SAR indicated favorable effects of the 5-chloro-2-thienyl P1 group and para-fluoro substitution within the phenylpiperazine P4 region, while substitution of the central anthranilamide ring further modulated activity [4].

QSAR modeling can convert such compact congeneric datasets into quantitative medicinal-chemistry hypotheses, provided that model complexity is carefully constrained, and goodness-of-fit is interpreted alongside cross-validation, Y-randomization, and applicability-domain analysis [8,9,10]. Multivariate adaptive regression splines (MARS) are especially useful in this setting because they retain an explicit mathematical form while allowing threshold-dependent and, when justified, interaction-dependent relationships between descriptors and biological activity [11,12]. Our previous integrated FXa study demonstrated the practical value of combining DFT-derived structures, descriptor filtering, Random Forest preselection, MARSplines modeling, applicability-domain assessment, model-guided analog design, and receptor-based prioritization [8].

The rationale for the present study was to construct a stepwise computational prioritization workflow in which each stage addressed a specific limitation of the preceding one. The piperazinylanthranilamide series reported by Khadse et al. was selected because it provides a congeneric dataset generated within a single experimental study and contains systematic structural variation across the P1, central-ring, and P4 regions. Rivaroxaban was excluded from model training because it does not belong to this congeneric scaffold series. DFT-optimized geometries and alvaDesc descriptors were used to provide a standardized three-dimensional structural representation, while Random Forest preselection reduced the initially high-dimensional descriptor space. Given the limited dataset size, MARSplines model complexity was deliberately constrained, and applicability-domain assessment was treated as an integral component of prospective prediction to reduce the risk of overinterpreting extrapolative estimates.

Prospective design retained the 5-chloro-2-thienyl P1 group and the central-ring environments represented by the highly active compounds **6y** and **7f**, while exploring meta-substitution and controlled multi-substitution within the phenylpiperazine P4 region, which were less represented in the source series. Compounds were advanced to receptor-based evaluation primarily according to predicted activity and applicability-domain status. Compounds **6y** and **7f** were included as experimentally characterized same-series references, whereas apixaban served as the crystallographic pose-recovery comparator. An initial 100 ns MD simulation was performed for each of the thirteen FXa–ligand complexes to examine the dynamic behavior of the docking poses, and PAX-M09, PAX-M15, and PAX-M18 were subsequently selected for independent replicate simulations on the basis of the initial structural and interactional screening before final MM/GBSA analysis. SwissADME, BOILED-Egg, and pkCSM profiling were additionally included so that prospective prioritization considered predicted developability liabilities alongside ligand-based and receptor-based results. Thus, the individual computational stages were used as complementary sequential filters rather than as independent evidence of experimentally confirmed activity.

## 2. Results

### 2.1. Activity Distribution and Source-Series SAR

The twenty-compound regression dataset covered an approximately 1.82-log-unit pIC_50_ interval, from 4.399 for **8d** (IC_50_ = 39.9 µM) to 6.215 for **6y** (IC_50_ = 0.61 µM). The upper end of the distribution was defined by **6y**, **7f** (pIC_50_ = 6.131), and **7v** (pIC_50_ = 5.745), whereas most remaining derivatives occupied the pIC_50_ range of approximately 4.45–5.20. This distribution provides a meaningful, although relatively small, range of responses for regression modeling.

The source-series SAR is consistent with a major role of the P1/P4 combination. Within the 5-chloro-2-thienyl subsets, para-fluoro substitution at the phenylpiperazine P4 region generated the most active compounds on both the monomethoxy (**6y**) and dimethoxy (**7f**) cores. The 5-chloro-2-thienyl P1 fragment generally produced more favorable activity than the corresponding 6-chloro-3-pyridyl or 4-chlorophenyl groups, while chlorination of the central anthranilamide ring was less favorable overall. These trends provided the medicinal-chemistry rationale for retaining the 5-chloro-2-thienyl P1 group in the subsequent design panel.

### 2.2. DFT Optimization and Geometry Quality Control

All twenty training compounds completed the Gaussian optimization/frequency workflow with normal termination. Each optimized structure showed zero imaginary frequencies, confirming local-minimum geometries at the applied level of theory. Formula checks reconstructed from the final coordinates were consistent with the expected molecular formulas for all compounds. The lowest minimum vibrational frequency observed across the set was 6.7408 cm^−1^. These checks supported the use of the final DFT geometries as a consistent structural basis for 3D descriptor calculation.

### 2.3. Descriptor Reduction and Random Forest Ranking

The raw alvaDesc calculation yielded 5799 molecular descriptors per compound. Technical reduction retained 1102 descriptors, and manual exclusion of three additional 95%-constant variables resulted in 1099 descriptors entering the Random Forest ranking. The most stable high-ranking variables included GATS4e, Mor21m, RDF040p, RDF040s, GATS3s, RDF140s, Mor31charge, Mor26s, Mor07s, RDF055e, GATS8p, and PNSA-3. After stricter correlation pruning of the high-importance variables, a 30-descriptor matrix was retained for regression-model comparison. Notably, Mor21m and RDF055e, which later entered the final MARSplines model, were already prominent in the supervised ranking, whereas SHED_AL emerged as useful in the multivariate nonlinear combination.

### 2.4. Comparative Evaluation of Regression Algorithms

The best three-descriptor MLR model used RDF055e, GATS4s, and Kp, achieving R^2^ = 0.668 and Q2_LOO = 0.533. Adding a fourth linear descriptor increased R^2^ to 0.741 but provided only a marginal gain in Q2_LOO (0.544) and increased model complexity. PLS, ridge regression, elastic net, and SVR variants showed lower cross-validated performance than the selected MLR baselines. By contrast, the constrained additive MARSplines model provided a marked improvement while retaining the same number of active descriptor terms as the three-variable MLR model (Table 1).

For reference, the best three-descriptor MLR equation (Equation (1)) was:

(1) pIC_50_ = 7.93935 + 0.10730(RDF055e) − 2.97833(GATS4s) − 1.88342(Kp).

Although this MLR model preserved the activity ranking reasonably well, it substantially underpredicted several of the most active compounds. It produced a larger cross-validated error than the final nonlinear model.

### 2.5. Final Constrained Additive MARSplines Model

The selected model was a three-term additive MARSplines equation. The final specification retained one descriptor from the 3D-MoRSE family (Mor21m), one electronegativity-weighted radial-distribution-function descriptor (RDF055e), and one Shannon-entropy pharmacophore-distribution descriptor (SHED_AL) (Table 2). The model is given by Equation (2):

(2) pIC_50_ = 4.41730663 + 5.63720499(BF1) + 0.16258023(BF2) + 0.59725598(BF3),

where the active basis functions are defined as follows (Equations (3)–(5)):

(3) BF1 = max(0, Mor21m + 0.95363491),

(4) BF2 = max(0, RDF055e − 16.71696820),

(5) BF3 = max(0, 11.37792108 − SHED_AL).

The three positive coefficients do not imply that unrestricted increases in the corresponding raw descriptors are universally favorable; rather, each term contributes only on one side of its fitted knot. BF1 indicates that mass-weighted 3D-MoRSE information becomes positively associated with activity once Mor21m exceeds approximately −0.954. BF2 introduces a positive contribution when RDF055e exceeds approximately 16.72, indicating a threshold dependence on the electronegativity-weighted three-dimensional interatomic-distance distribution. BF3 contributes when SHED_AL falls below approximately 11.38, linking activity to the entropy/distribution of acceptor-lipophilic pharmacophore relationships. These associations should be interpreted as statistical predictors of structure–activity relationships rather than as direct mechanistic interaction energies. In practical terms, Mor21m, RDF055e, and SHED_AL are numerical representations of molecular structural features whose values are associated with changes in experimental pIC_50_ within the modeled chemical series. Their contributions to the predicted response are determined by the fitted MARSplines hinge functions and apply only on the relevant side of the corresponding descriptor thresholds. They do not represent binding-energy components, the strength of individual intermolecular interactions, or direct measurements of specific protein–ligand contacts.

### 2.6. Internal Validation and Applicability Domain

The final additive MARSplines model explained 90.4% of the variance in the training response (R^2^ = 0.904), with an adjusted R^2^ = 0.886, RMSEC = 0.158, and a calibration MAE of 0.116 pIC_50_ units. Leave-one-out cross-validation yielded Q^2^_LOO = 0.846, RMSECV_LOO = 0.200, and MAE_LOO = 0.149. Because leave-one-out estimates may be optimistic for a small dataset, model robustness was additionally examined using 200 repeated five-fold partitions and 1000 Y-randomization trials. Repeated five-fold cross-validation yielded a mean Q^2^ of 0.830 ± 0.041, with mean RMSE and MAE values of 0.209 and 0.156, respectively. In the Y-randomization analysis, the mean randomized Q^2^ was −0.377 and the maximum was 0.603; none of the 1000 randomized models reached the observed Q^2^_LOO of 0.846. These results support the non-random character and internal robustness of the fitted relationship, while not constituting external validation (Table 3). Figure 1 shows experimental versus leave-one-out-predicted pIC_50_ values, providing a direct visualization of internal prediction rather than calibration fit.

For the final three-term model, the leverage warning threshold was h* = 0.60. Compound **6y** showed leverage h = 0.611 and was therefore identified as a slightly high-leverage observation. This is chemically plausible because **6y** is also the most active compound and occupies an influential region of the modeled descriptor space. No compound exceeded the absolute standardized-residual threshold of 3. Compound **7m** was the closest to this limit (standardized residual = −2.987), indicating that it should be treated as a borderline response observation rather than automatically excluded. The training-set ranges of the three final descriptors were Mor21m = −1.257 to −0.796, RDF055e = 13.331 to 21.550, and SHED_AL = 10.376 to 13.222 (Figure 2).

### 2.7. Model-Guided Design Panel

The selected design panel comprised twenty analogs organized as ten matched P4 substitution pairs on the **6y**-like monomethoxy and **7f**-like dimethoxy cores (Figure 3). The design therefore probes positional and electronic effects while preserving the 5-chloro-2-thienyl P1 fragment associated with the most favorable source-series potency. The nearest-reference Morgan similarities were 0.754–0.867 for the monomethoxy series and 0.750–0.864 for the dimethoxy series, supporting their use as a focused, relatively conservative model-guided design set (Table 4).

### 2.8. Prospective DFT Optimization and Descriptor Calculation

All twenty PAX-M01-PAX-M20 calculations completed with normal Gaussian termination, successful optimization, and a stationary-point designation. No imaginary frequencies were detected for any prospective analog. The lowest minimum frequency in the panel was 4.810 cm^−1^ for PAX-M10; all remaining minimum frequencies were also positive. Atom counts, atom order, and molecular formulae matched the intended structures, and a final optimized geometry was extracted for every analog. These geometries were used directly for the prospective alvaDesc calculation. No descriptor reduction or supervised selection was repeated on the prospective set; only the three descriptors fixed during training-model development were extracted for prediction.

### 2.9. Prospective Activity Prediction and Applicability Domain

Applying the fixed MARSplines equation produced a wide range of raw pIC_50_ estimates, but the applicability-domain analysis substantially changed their interpretation. Seven analogs (PAX-M09, PAX-M02, PAX-M19, PAX-M14, PAX-M18, PAX-M12, and PAX-M04) satisfied both the leverage and descriptor-range criteria and were classified as strict in-domain. PAX-M15 had acceptable leverage (h = 0.527) but a SHED_AL value of 10.300, slightly below the training minimum of 10.376, and was therefore classified as borderline. The remaining twelve compounds were outside the domain. Several of these, most notably PAX-M10 and PAX-M06, generated apparently very favorable raw pIC_50_ values but had extreme leverage values (3.93 and 6.81, respectively); these estimates were treated as extrapolations rather than quantitative activity predictions (Table 5).

Among the strict in-domain compounds, PAX-M09 (3-Cl-4-F, **6y**-like monomethoxy core) was the strongest prospective prediction, with pIC_50_ = 6.001 and IC_50_ ≈ 1.00 µM. The next strict in-domain compounds were PAX-M02 (pIC_50_ = 5.224), PAX-M19 (5.169), PAX-M14 (5.119), and PAX-M18 (5.081). PAX-M15 was numerically stronger than PAX-M09 (pIC_50_ = 6.356; IC_50_ ≈ 0.44 µM) but remained borderline because of its small descriptor-range violation. Importantly, no strict in-domain prospective analog was predicted to exceed the experimental pIC_50_ values of the training-set leaders **6y** (6.215) and **7f** (6.131). Figure 4 illustrates the prospective predictions, their domain classifications, and the experimental activity levels for **6y** and **7f**.

### 2.10. Docking-Panel Selection

A ten-compound receptor-based panel was defined after combining prospective potency with applicability-domain status. The seven strict in-domain analogs were retained as the primary QSAR-supported set. PAX-M15 was retained as a borderline high-priority analog because its descriptor-range violation was small and its leverage remained below h*. PAX-M17 and PAX-M08 were selected as exploratory comparators because they were the most useful near-domain outside-domain candidates among the remaining designs, with leverage values of 0.838 and 0.670, respectively. This strategy deliberately excluded highly extrapolative compounds such as PAX-M06 and PAX-M10 despite their favorable raw pIC_50_ values (Table 6).

Compounds **6y** and **7f** were docked alongside the ten prospective analogs as experimental-series reference compounds, with apixaban included as the crystallographic binding-site and docking-validation reference. The SwissADME/BOILED-Egg and pkCSM profiles are presented in Section 2.11, and the molecular docking results are reported in Section 2.12.

### 2.11. In Silico ADMET and BOILED-Egg Profiling

SwissADME predicted high GI absorption and no BBB permeation for all ten PAX candidates, **6y**, **7f**, and rivaroxaban [13]. The designed PAX compounds remained substantially more lipophilic than rivaroxaban: consensus LogP values ranged from 3.92 (PAX-M14) to 4.89 (PAX-M19), compared with 2.25 for rivaroxaban. ESOL LogS values for the PAX set ranged from −6.80 to −6.03; all were classified as poorly soluble, whereas rivaroxaban showed an ESOL LogS of −4.00. Veber and Egan criteria were satisfied throughout the designed set, and the SwissADME bioavailability score was 0.55 for all compounds. PAX-M02 and PAX-M04 had no Lipinski violations, whereas most higher-molecular-weight analogs had one violation due to MW > 500. PAX-M18 and PAX-M08 each generated one PAINS alert (anil_di_alk_C), whereas the remaining PAX compounds showed no PAINS alerts and no Brenk alerts (Table 7).

The BOILED-Egg visualization reinforced the SwissADME permeability classification (Figure 5). All designed PAX analogs and both source-series controls clustered within the white region associated with favorable intestinal absorption, while remaining outside the yellow BBB-permeant region. These compounds were plotted as P-gp-negative points by SwissADME, whereas rivaroxaban was represented as a P-gp substrate. The strong contrast with the pkCSM P-gp call, which classified every molecule as a P-gp substrate and as a class I and class II P-gp inhibitor, was therefore treated as an explicit model-dependent disagreement rather than as a resolved mechanistic conclusion.

pkCSM independently predicted favorable absorption for the series, with Caco-2 values of 1.002–1.155 and human intestinal absorption of 89.4–94.5% across the ten PAX candidates. Predicted skin-permeability values were narrowly distributed (−2.802 to −2.736). Distribution estimates suggested low-to-moderate VDss (−0.139 to 0.140) and very low free fractions (0–0.023) for the PAX compounds, consistent with extensive plasma-protein binding. BBB log values were negative for all candidates (−0.814 to −0.290), and CNS-permeability outputs ranged from −3.005 to −1.805, with PAX-M18 and PAX-M15 showing the lowest predicted CNS penetration. All thirteen molecules were predicted to be non-substrates of renal OCT2.

The pkCSM metabolism panel yielded a nearly uniform scaffold-level pattern. All PAX analogs, **6y**, and **7f** were predicted as CYP3A4 substrates and as inhibitors of CYP2C19, CYP2C9, and CYP3A4, while none were predicted as a CYP2D6 substrate, CYP1A2 inhibitor, or CYP2D6 inhibitor. Rivaroxaban shared the CYP3A4-substrate, CYP2C19-inhibitor, and CYP3A4-inhibitor calls but was not predicted to inhibit CYP2C9. These pkCSM assignments only partly agreed with SwissADME, particularly for CYP2D6 and CYP3A4 inhibition, again indicating model-dependent uncertainty. Predicted total clearance across the PAX series ranged from 0.308 (PAX-M02) to 0.790 (PAX-M14), with PAX-M09 at 0.347 and rivaroxaban at 0.298 (Table 8).

No compound was predicted to be AMES-positive or a skin sensitizer, and all were classified as hERG-I non-inhibitors. Conversely, every molecule, including rivaroxaban, was classified as a hERG-II inhibitor. Within the PAX series, acute rat LD50 model values ranged from 2.259 to 2.393 and chronic LOAEL values from 1.111 to 1.714; PAX-M15 had the highest predicted maximum tolerated dose (0.157) and chronic LOAEL (1.714), whereas PAX-M17 had the highest acute LD50 model value (2.393). Hepatotoxicity was predicted for all PAX compounds except PAX-M12. T. pyriformis toxicity values ranged from 0.291 to 0.395, and minnow-toxicity outputs from −1.344 to −0.389 for the PAX set; corresponding ecological-toxicity values were not returned for rivaroxaban in the supplied pkCSM output.

Integrated consideration of QSAR confidence and the two ADMET platforms did not identify a single uniformly optimal molecule. PAX-M09 remained the primary efficacy-oriented lead because it was the strongest strict in-domain QSAR prediction, but its high lipophilicity, poor solubility, and predicted metabolic/hepatotoxicity liabilities require caution. PAX-M19 provided a more balanced, strict in-domain profile with fewer SwissADME CYP inhibition calls, although its molecular weight and lipophilicity remained high. PAX-M15 combined the strongest retained prospective pIC_50_ estimate with favorable pkCSM tolerated-dose/LOAEL outputs and limited CNS penetration, but remained borderline in the QSAR applicability domain. PAX-M02 offered the cleanest conventional drug-likeness profile among strict in-domain designs (zero Lipinski violations and zero PAINS alerts), whereas PAX-M12 was notable as the only PAX compound predicted by pkCSM as non-hepatotoxic despite its weaker QSAR potency. These complementary profiles motivated the retention of PAX-M09, PAX-M19, PAX-M15, and PAX-M02 as the most informative candidates for receptor-based follow-up, with PAX-M12 as a safety-oriented comparator.

### 2.12. Molecular Docking Studies

Redocking of apixaban into human factor Xa (FXa; PDB ID: 2P16) reproduced the crystallographic ligand conformation with an RMSD of 0.953 Å (Figure 6A), consistent with the reference-ligand pose recovery reported in the previous FXa study [8]. This result supports reproduction of the apixaban binding mode in the selected receptor structure (Figure 6B); it does not, by itself, validate affinity ranking across the PAX series. The apixaban interaction diagram showed hydrogen bonds involving Gly216 and Glu146, together with water-associated contacts, including a network involving Ser214.

Apixaban gave the most favorable docking score in the thirteen-compound panel (−11.362). Scores for the ten designed analogs ranged from −10.267 for PAX-M17 to −9.039 for PAX-M04 (Table 9). PAX-M17 and PAX-M19 (−10.223) occupied the first two positions among the PAX compounds, with a numerical separation of only 0.044 score units. Their scores were also close to that of the same-series reference **6y** (−10.086). PAX-M09 (−9.952) and PAX-M12 (−9.910) formed another closely scored pair. These numerical rankings were not interpreted as resolved differences in experimental inhibitory potency.

The recurring residue neighborhoods in the two-dimensional interaction diagrams were consistent with accommodation of the common scaffold across the canonical S1/S4 binding region (Figure 7 and Figure 8). The 5-chlorothiophene fragment was associated with the Asp189/Ala190/Cys191/Gln192 region, whereas the terminal substituted phenyl group was positioned in the hydrophobic/aromatic environment associated with Tyr99, Phe174, and Trp215. Residues shown near a ligand were distinguished from residues connected to it by an explicit directional-interaction annotation. A recurrent hydrogen-bonding motif involved the thiophene-linked amide NH and Gly216, along with an interaction between Gly218 and the carbonyl that links the central aromatic ring to the piperazine.

PAX-M19 combined the Gly216/Gly218 motif with an additional hydrogen bond involving Gln192 and a core methoxy oxygen, as well as an explicitly annotated π–π interaction between Trp215 and the terminal 3-chloro-4-fluorophenyl ring. PAX-M17 also displayed hydrogen bonds to Gly216 and Gly218, but its diagram did not contain an explicit π–π annotation. Thus, their nearly identical scores were associated with distinguishable contact patterns. PAX-M09 retained the Gly216/Gly218 and Trp215 contacts, whereas PAX-M12 additionally displayed the Gln192-associated methoxy interaction.

The reference compounds provided a further instructive contrast. The **6y** diagram showed a Gly218 hydrogen bond and a Trp215 π–π contact, while **7f** displayed hydrogen bonds involving Gly216, Gly218, and Gln192 without an explicit π–π annotation. Nevertheless, **6y** scored more favorably than **7f** (−10.086 versus −9.331). Similarly, PAX-M04 retained both glycine-associated hydrogen bonds and a Trp215 π–π contact despite giving the least favorable PAX score. The number of annotated hydrogen bonds or aromatic contacts, therefore, did not reproduce the docking-score order.

Additional differences were apparent among the remaining analogs. PAX-M02 displayed the Gly216/Gly218 and Trp215 motif, while PAX-M14 showed hydrogen bonds involving Gly216, Gly218, and Gln192. PAX-M15 and PAX-M18 retained a Gly216 hydrogen bond, but neither diagram explicitly annotated a Gly218 hydrogen bond or a π–π contact. PAX-M08 combined a Gly216 hydrogen bond with aromatic contacts involving Trp215 and Phe174.

### 2.13. Molecular Dynamics and MM/GBSA Analysis

#### 2.13.1. Initial 100 ns Screening

Nineteen nominal 100 ns trajectories were available: one initial run for each of the thirteen complexes, followed by two additional runs for each of PAX-M09, PAX-M15, and PAX-M18. Initial-run RMSD profiles and contact-count distributions are shown in Supplementary Figures S1 and S2. Table 10 summarizes the initial screen, whereas Table 11 and Supplementary Figures S3 and S4 describe the selected replicates.

Protein backbone RMSD remained relatively limited across the initial runs, with 50–100 ns means of 1.19–1.60 Å. Apixaban showed a protein-aligned ligand RMSD of 0.90 ± 0.20 Å and a ligand self-aligned RMSD of 0.43 ± 0.10 Å. Several PAX compounds remained closer to their respective starting configurations, including PAX-M15 (1.59 ± 0.33 Å), PAX-M04 (2.11 ± 0.39 Å), PAX-M09 (2.18 ± 0.39 Å), and PAX-M18 (2.38 ± 0.28 Å). The initial PAX-M12 trajectory displayed a late change: its mean protein-aligned ligand RMSD increased from approximately 2.21 Å over 60–80 ns to 4.09 Å over 80–100 ns.

Larger positional deviations were observed for **6y**, **7f**, PAX-M02, PAX-M14, PAX-M17, and PAX-M19. These changes did not coincide with sustained loss of all exported protein contacts in the terminal interval. Over frames 800–1000, at least one protein contact, including water-mediated contacts, was recorded in 99.5–100% of frames for every run. Contact with the defined eight-residue binding-site set was retained in 94.0–100% of terminal frames (Supplementary Tables S1–S4). This supports the persistence of protein-associated configurations rather than a clear, sustained, complete separation, but does not prove the preservation of the original binding pose or resolve the three-dimensional pathway of the rearrangements.

#### 2.13.2. Reproducibility of the Selected PAX Complexes

PAX-M15 gave the most closely grouped positional RMSD values among the replicated PAX candidates: 1.59 ± 0.33, 2.11 ± 0.50, and 1.78 ± 0.43 Å in runs 1–3, respectively. The corresponding backbone means were 1.37, 1.35, and 1.30 Å. Its polar and water-mediated interaction pattern also recurred: Gly216 hydrogen-bond occupancy was 38.3%, 51.9%, and 48.9%, Gly218 occupancy was 64.7%, 49.1%, and 51.9%, and Ser195 water-bridge occupancy was 70.9%, 74.5%, and 71.3%. Trp215 π–π occupancy varied more substantially (80.2%, 55.7%, and 44.5%), indicating that comparable global RMSD did not require identical aromatic-contact populations.

PAX-M09 showed greater between-run heterogeneity. Protein-aligned ligand RMSD was 2.18 ± 0.39 and 2.65 ± 0.38 Å in runs 1 and 3, but 5.79 ± 0.20 Å in run 2. Runs 1 and 3 retained high Trp215 π–π occupancy (93.4% and 98.4%), whereas no such contact was recorded in the 50–100 ns interval of run 2. Instead, run 2 exhibited high Gly216 and Gly218 hydrogen-bond occupancies (86.8% and 89.8%). These observables support a distinct positional and interaction pattern in the second run. They do not, without coordinate-based clustering or free-energy analysis, establish two thermodynamically characterized binding states.

PAX-M18 also retained protein contacts but displayed variable positional behavior, with protein-aligned ligand RMSD values of 2.38 ± 0.28, 3.50 ± 0.55, and 3.09 ± 0.56 Å. Gly218 hydrogen-bond occupancy changed from 25.1% to 80.6% and 16.4% across the three runs, while Ser195 water-bridge occupancy was 67.5%, 81.6%, and 32.7%. Full-trajectory Phe174 Cα RMSF was 1.90, 1.07, and 2.70 Å, respectively (Supplementary Table S2). Thus, the third run exhibited greater local flexibility in this region, without evidence of a comparable increase in global backbone displacement. These results distinguish PAX-M18 from the more consistent overall positional behavior of PAX-M15.

#### 2.13.3. MM/GBSA Endpoint Estimates

Run-resolved MM/GBSA estimates are reported in Table 9. Apixaban, for which one trajectory was available, yielded an estimate of −77.31 ± 3.42 kcal/mol. Among the three replicated PAX compounds, PAX-M15 consistently showed the most favorable MM/GBSA values across all three independent runs (−68.76 ± 3.21, −70.11 ± 3.76, and −69.42 ± 3.14 kcal/mol for Runs 1–3, respectively). PAX-M09 yielded −62.41 ± 3.86, −64.86 ± 4.42, and −62.06 ± 3.64 kcal/mol, whereas PAX-M18 yielded −60.13 ± 5.43, −62.48 ± 4.83, and −61.95 ± 5.32 kcal/mol. Thus, the same energetic ordering, PAX-M15 followed by PAX-M09 and PAX-M18, was observed in each of the three runs. Importantly, the run-resolved presentation retains the energetic variability alongside the structural and interactional heterogeneity observed between independent trajectories. This is a comparison of the computational endpoint estimates and does not demonstrate improved experimental inhibitory potency.

For the PAX compounds represented by a single trajectory, MM/GBSA estimates ranged from −54.81 ± 5.01 kcal/mol for PAX-M12 to −42.85 ± 3.06 kcal/mol for PAX-M17. PAX-M17 and PAX-M19, the two highest-scoring PAX ligands in docking, yielded substantially less favorable endpoint estimates (−42.85 ± 3.06 and −48.73 ± 6.42 kcal/mol, respectively) than the run-specific estimates for the replicated subset. Conversely, PAX-M15 did not rank highest in docking but consistently yielded the most favorable MM/GBSA estimates across all three independent runs.

## 3. Discussion

The present study suggests that nonlinear threshold-dependent relationships are important for describing FXa inhibition within this compact piperazinylanthranilamide series. The advantage of the selected additive MARSplines model over the evaluated linear alternatives is therefore not simply a matter of improved numerical fit, but rather its ability to represent local changes in structure–activity relationships using a small number of interpretable hinge functions. At the same time, the limited size and chemical scope of the dataset mean that the model should be regarded as a local representation of this specific congeneric series rather than as a general predictor of FXa inhibition.

The three retained descriptors point to a combined role of molecular shape, interatomic-distance organization, and pharmacophore distribution. Mor21m and RDF055e encode geometry-dependent three-dimensional information, whereas SHED_AL describes the distributional entropy of acceptor–lipophilic pharmacophore relationships. These variables should therefore be interpreted as statistical predictors of structure–activity relationships rather than as direct mechanistic interaction energies. Their fitted contributions indicate which regions of descriptor space are associated with higher experimental pIC_50_ values within the present series, but they do not represent individual hydrogen-bond strengths, binding-energy components, or specific protein–ligand contacts. Because Mor21m and RDF055e are conformationally dependent, their numerical values are also conditional on the selected DFT-optimized conformer. This is particularly relevant to hinge-based models, in which relatively small conformationally induced descriptor changes near a fitted threshold may alter the contribution of a basis function. Ensemble-based descriptor analysis would therefore represent a useful future extension [15,16].

One of the most important practical findings is the role of applicability-domain analysis in prospective prioritization. Several newly designed compounds received numerically favorable raw predictions while lying outside the modeled descriptor space. Treating such values as equivalent to in-domain predictions would have created an artificial impression of strongly improved potency. The distinction between strict in-domain, borderline, and extrapolative predictions therefore had a direct influence on candidate selection. In this context, PAX-M09 and PAX-M15 illustrate two complementary situations: the former represents the strongest strict in-domain prediction, whereas the latter combines a favorable numerical prediction with a small descriptor-range violation. This distinction is more informative for prospective decision-making than ranking compounds solely by predicted pIC_50_.

The receptor-based analyses provided an additional and partly independent perspective on the ligand-based ranking. The absence of complete agreement among QSAR, docking, MD, and MM/GBSA should not be interpreted as a methodological inconsistency, because these approaches describe different aspects of the system. QSAR relates molecular descriptors to experimental activity within the training series, docking evaluates static pose complementarity and scoring within a fixed receptor structure, whereas MD and MM/GBSA examine the behavior and approximate energetics of trajectory-derived configurations. The fact that the highest docking scores did not necessarily correspond to the most favorable dynamic or MM/GBSA behavior therefore highlights the value of sequential rather than single-score prioritization.

The replicated MD simulations further emphasize that similar endpoint energy estimates do not necessarily imply identical structural behavior. PAX-M15 showed the most consistent overall receptor-based profile among the replicated candidates, whereas PAX-M09 and PAX-M18 displayed greater trajectory-to-trajectory heterogeneity. PAX-M09 nevertheless remains particularly relevant because of its strict applicability-domain status, while PAX-M15 represents a promising but borderline QSAR prediction supported by comparatively consistent receptor-based behavior. PAX-M18 provides an additional in-domain candidate with a distinct dynamic profile. These differences support considering the three compounds as complementary experimental priorities rather than as a simple numerical ranking.

The same-series reference compounds and apixaban serve different interpretive purposes. Compounds **6y** and **7f** provide chemically matched examples with experimentally measured FXa activity, whereas apixaban provides a crystallographic pose-recovery reference. The apixaban redocking result therefore supports the ability of the docking protocol to reproduce the experimentally observed binding pose but does not constitute quantitative validation of Glide XP scoring for the new piperazinylanthranilamide analogs. The identical pose-recovery result obtained previously with the same receptor workflow supports procedural reproducibility [8], while the limited number of same-series experimental references prevents formal validation of a docking-score/activity relationship.

Developability considerations also modify the interpretation of potency-oriented prioritization. The predicted combination of relatively high lipophilicity, limited aqueous solubility, low unbound fraction, and potential CYP liabilities indicates that further optimization should not focus exclusively on receptor affinity. The disagreement between SwissADME and pkCSM for P-gp and selected CYP classifications further illustrates that these platforms are best used as complementary screening tools rather than definitive mechanistic assays. Once prioritized PAX analogs are synthesized, an initial experimental ADME panel should include solubility, bidirectional Caco-2 or MDCK-MDR1 permeability, human liver microsomal stability, major CYP inhibition assays, and plasma-protein binding measurements. Such data would help distinguish whether favorable computational prioritization can be translated into a practically developable chemical series.

Several limitations should be considered when interpreting the present results. The QSAR model was developed from only twenty compounds belonging to a single literature series, and the reported resampling statistics represent internal robustness rather than validation against an independent external chemical set. Descriptor calculations were based on a single DFT-optimized conformer for each compound and therefore do not capture full conformational ensembles. The newly designed PAX analogs have not yet been synthesized or experimentally tested, and the SwissADME and pkCSM results should be regarded as screening predictions rather than measured ADME properties. The receptor-based analyses are additionally limited by finite simulation times and unequal replication, with late-stage positional changes observed for some trajectories. Finally, MM/GBSA provides approximate comparative endpoint estimates and does not fully account for all entropic and solvent contributions. Accordingly, the final prioritization should be viewed as hypothesis-generating and requires subsequent experimental validation.

## 4. Materials and Methods

### 4.1. Dataset Preparation

The regression dataset consisted of twenty piperazinylanthranilamide derivatives reported by Khadse et al. for which experimental FXa IC_50_ values were available [4]. Rivaroxaban, included as a reference compound in the source study, was not included in model training because it does not belong to the investigated congeneric series. The dependent variable was pIC_50_, calculated from the mean IC_50_ value expressed in micromolar units according to Equation (6):

(6) pIC_50_ = 6 − log10(IC_50_ [µM]).

This transformation was used so that higher pIC_50_ values corresponded to greater FXa inhibitory potency. The structural coding of the dataset retained the source-study P1, central-ring (X), and phenylpiperazine P4 (Y) substituent definitions. The complete dataset is summarized in Table 12, and the corresponding structures are shown in Figure 9.

### 4.2. Three-Dimensional Structure Generation and DFT Geometry Optimization

Three-dimensional starting structures were consistently prepared for all 20 compounds before quantum-chemical optimization. For each molecule, 80 initial conformers were generated using the ETKDGv3 distance-geometry procedure. The conformers were minimized using the MMFF94s force field, and the lowest-energy converged conformer was selected as the starting geometry. This automated conformer-selection step replaced manual construction of starting geometry while retaining a common protocol across the entire dataset.

Final geometry optimization and vibrational frequency calculations were performed using Gaussian 16 [17]. All compounds were treated as neutral singlets and optimized using the B3LYP hybrid density functional [18,19,20] with the 6-311++G(d,p) basis set [21,22,23]. The route section was B3LYP/6-311++G(d,p) Opt Freq. Frequency calculations were performed at the same level of theory to confirm that the optimized structures corresponded to local minima and contained no imaginary vibrational frequencies. The final optimized coordinates, rather than re-generated 3D conformations, were used for molecular descriptor calculation.

### 4.3. Molecular Descriptor Calculation and Technical Variable Reduction

Molecular descriptors were calculated from the Gaussian-optimized geometries using alvaDesc V3.0.16 (Alvascience srl, Lecco, Italy) [24]. The optimized three-dimensional coordinates were retained, and no additional 3D coordinate generation or force-field optimization was applied in alvaDesc. Molecular descriptors were calculated across the available descriptor families, whereas fingerprint variables were excluded from the present regression workflow.

The initial descriptor matrix contained 5799 molecular descriptors. Objective technical reduction was first performed in alvaDesc by removing constant descriptors; descriptors with more than 95% constant values; descriptors with a standard deviation below 0.0001; descriptors with at least one missing value; and one member of descriptor pairs with an absolute correlation greater than or equal to 0.95. Two descriptors with values missing for all compounds (Psi_e_1d and Psi_e_1s) were removed in this process. The alvaDesc reduction retained 1102 descriptors. Because a descriptor identical in 19 of 20 compounds is exactly 95% constant and therefore is not removed by a strict “greater than 95%” rule, three additional variables (Eta_D_AlphaA, B03[N-Cl], and CATS3D_11_AA) were excluded manually as near-constant descriptors. The final matrix entering supervised preselection contained 1099 descriptors.

### 4.4. Random Forest-Based Supervised Descriptor Preselection

Random Forest regression was used as a supervised descriptor-ranking method with pIC_50_ as the response variable [25]. Descriptor importance was aggregated across repeated fits, and the most stable variables were identified using mean importance, mean rank, and frequency of occurrence among the top-ranked descriptors. The resulting Top-100 list was further pruned via pairwise correlation with a stricter absolute correlation threshold of 0.85, yielding a 30-descriptor candidate matrix for comparative regression modeling. Random Forest was used only for variable ranking, not as the final predictive model, because its out-of-bag predictive performance was weak on this very small dataset.

### 4.5. Comparative Regression Modeling

Multiple linear regression (MLR) was first used to establish parsimonious linear baselines. Candidate models containing three or four descriptors were screened, and model choice was based on a combination of goodness-of-fit, leave-one-out predictivity, prediction error, and multicollinearity. In parallel, partial least-squares regression (PLS), ridge regression, elastic-net regression, linear support-vector regression (SVR), and radial-basis-function SVR were evaluated as alternative machine-learning approaches. Hyperparameter-dependent alternatives were assessed with nested internal resampling to reduce optimistic tuning bias.

Nonlinear modeling was then performed using a constrained additive MARSplines strategy [11,12]. A hinge basis function was defined as h(x) = max(0,x), with the knot determining the descriptor threshold above or below which a term contributes to the predicted response. Because the dataset comprised only twenty compounds, model complexity was deliberately restricted to no more than four active terms. In addition, each candidate hinge had to be supported by at least five training compounds, preventing the final model from being dominated by thresholds that are active only for one or two extreme observations. The final model was expressed explicitly as a hinge-basis regression, and coefficient estimates were obtained by ordinary least squares using Python (version 3.14.8.)-based numerical and machine-learning libraries [26].

To make the model-construction sequence explicit, the workflow proceeded from 5799 initially calculated alvaDesc descriptors through technical filtering to 1102 variables, followed by removal of three additional near-constant descriptors, leaving 1099 variables for supervised ranking. Random Forest preselection generated a Top-100 descriptor set, which was subsequently reduced by pairwise correlation pruning at |r| ≥ 0.85 to a 30-descriptor candidate matrix. Comparative regression modeling was then performed using this reduced matrix. For MARSplines, model complexity was constrained to no more than four active basis-function terms, and each candidate hinge was required to be supported by at least five training compounds. Candidate models were evaluated using goodness-of-fit, cross-validated predictive performance, prediction-error statistics, parsimony, and multicollinearity diagnostics. All descriptor-selection and model-selection steps were performed exclusively using the original 20-compound experimental dataset. After selection, the final MARSplines specification was fixed and subsequently applied to the prospective PAX analogs without descriptor reselection or model refitting.

### 4.6. Model Validation

Goodness-of-fit was characterized by R^2^, adjusted R^2^, root-mean-square error of calibration (RMSEC), and calibration mean absolute error (MAE). Internal predictive performance was evaluated by leave-one-out cross-validation using Q2_LOO, RMSECV_LOO, MAE_LOO, and PRESS. Ranking behavior was additionally characterized by Spearman correlation between experimental and predicted activities. To examine stability across alternative sample partitions, repeated five-fold cross-validation was performed for 200 randomized partitions. For this robustness analysis, the final basis-function specification was fixed, and model coefficients were refitted within each training fold.

Y-randomization was performed using 1000 random permutations of the pIC_50_ response while retaining the final descriptor/basis matrix [9,10,27,28]. For each permutation, model coefficients were refitted, and the resulting R^2^ and Q2_LOO values were calculated. The number of randomized models that reached or exceeded the observed Q2_LOO was recorded. Variance inflation factors (VIFs) were calculated to evaluate multicollinearity among the final basis functions. Because descriptor and hinge selection preceded these internal validation procedures, the reported resampling statistics are interpreted as internal robustness estimates and do not substitute for validation on a genuinely external chemical series.

### 4.7. Applicability Domain

The applicability domain (AD) of the final model was assessed using leverage and standardized residuals. The warning leverage was calculated as h* = 3(k + 1)/n, where k is the number of active predictor terms and n is the number of training compounds [9,27]. For the selected three-term model, h* was 0.60. Compounds with leverage above h* were treated as structurally influential, whereas observations with absolute standardized residuals greater than 3 were considered response outliers. The practical descriptor-domain ranges of Mor21m, RDF055e, and SHED_AL in the training set were also recorded and used to evaluate the newly designed analogs.

### 4.8. Model-Guided Design and Prospective DFT/AlvaDesc Calculation

A focused set of twenty new piperazinylanthranilamide analogs (PAX-M01-PAX-M20) was designed after selection of the final QSAR model. The 5-chloro-2-thienyl P1 fragment was retained because it was associated with the most favorable source-series activity profile, and two central-ring environments were selected to remain close to the high-activity compounds **6y** and **7f**: a **6y**-like monomethoxy core and a **7f**-like dimethoxy core. Ten P4 phenylpiperazine substitution patterns were explored in matched pairs across both cores: 3-F, 3-Cl, 3-Me, 3-OMe, 3,4-F2, 3,5-F2, 3-F-4-Me, 3-F-4-OMe, 3-Cl-4-F, and 3,4,5-F3.

The design intentionally emphasized meta substitution and controlled disubstitution, which were not represented in the twenty-compound regression subset and were less extensively explored in the source series than the corresponding ortho/para patterns. Morgan-fingerprint similarity to the nearest source reference (**6y** or **7f**) ranged from 0.750 to 0.867, providing a preliminary indication that the candidates remained relatively close to the modeled chemical space. Three-dimensional starting structures were generated with the same ETKDGv3/80-conformer/MMFF94s protocol used for the training compounds. Each prospective analog was then optimized as a neutral singlet using B3LYP/6-311++G(d,p), followed by a frequency calculation. Only normally terminated, stationary-point structures with zero imaginary frequencies were accepted. The final DFT geometries were exported to SDF and used directly for a full alvaDesc descriptor calculation without a second variable-reduction step.

### 4.9. Prospective MARS Prediction and Applicability-Domain Filtering

Prospective activity was calculated by extracting Mor21m, RDF055e, and SHED_AL from the complete alvaDesc export and applying the final fixed MARSplines equation without refitting or reselection. Predicted IC_50_ values were obtained from IC_50_ [µM] = 10^(6−pIC50)^. Applicability-domain assessment combined the leverage-warning threshold h* = 0.60 with the observed training ranges for Mor21m (−1.257 to −0.796), RDF055e (13.331 to 21.550), and SHED_AL (10.376 to 13.222). Predictions meeting both criteria were classified as strict in-domain. Compounds with acceptable leverage but a small descriptor-range violation were treated as borderline, whereas compounds with elevated leverage and/or more substantive range violations were classified as outside-domain and their raw pIC_50_ values were not interpreted quantitatively.

### 4.10. Molecular Docking Calculations

#### 4.10.1. Ligand Preparation

The receptor-based panel comprised ten prospectively designed PAX analogs selected after QSAR prediction and applicability domain assessment: PAX-M09, PAX-M02, PAX-M19, PAX-M14, PAX-M18, PAX-M12, PAX-M04, PAX-M15, PAX-M17, and PAX-M08. Compounds **6y** and **7f**, the two most active experimentally characterized members of the source piperazinylanthranilamide series, were included as experimental-series reference compounds. In contrast, apixaban was used as the crystallographic binding-site and docking-validation reference.

Ligand structures were prepared using the LigPrep module implemented in Schrödinger Maestro 2023-3 (Schrödinger, LLC, New York, NY, USA). The OPLS4 force field was applied during ligand preparation, and relevant ionization states were generated at physiological pH (7.4 ± 0.5). Low-energy prepared structures were retained for subsequent molecular docking calculations. This workflow was kept consistent with the previously reported factor Xa docking protocol [8].

#### 4.10.2. Protein Preparation

The crystal structure of human coagulation factor Xa (FXa) in complex with apixaban (PDB ID: 2P16) was used as the receptor structure. Protein preparation was performed using the Protein Preparation Wizard implemented in Schrödinger Maestro. Bond orders were assigned, hydrogen atoms were added, missing side chains were completed where necessary, and protonation states were optimized for physiological conditions. Crystallographic water molecules not directly involved in ligand recognition were removed. The prepared protein structure was subsequently subjected to restrained energy minimization using the OPLS4 force field.

#### 4.10.3. Docking Pose-Recovery Assessment

Before production docking, the co-crystallized apixaban molecule was extracted from the 2P16 structure and redocked into the FXa active site using the same docking protocol subsequently applied to the investigated compounds. The docked and crystallographic poses were compared by root-mean-square deviation (RMSD). The resulting redocked pose reproduced the crystallographic binding mode with an RMSD of 0.953 Å, demonstrating close recovery of the experimentally observed apixaban pose. This validation was used specifically to assess pose reproduction within the predefined FXa binding site and was not interpreted as quantitative validation of Glide XP scoring for the newly designed piperazinylanthranilamide analogs.

#### 4.10.4. Molecular Docking Procedure

The receptor grid was generated using the centroid of the co-crystallized apixaban molecule as the center of the FXa binding site. The inner ligand-diameter midpoint box was set to 10 Å × 10 Å × 10 Å and the outer bounding box to 30 × 30 × 30 Å, providing complete coverage of the canonical inhibitor-binding pocket. No positional, hydrogen-bonding, or metal-coordination constraints were applied.

Molecular docking calculations were performed using the Glide Extra Precision (XP) protocol implemented in Schrödinger Maestro. Default XP sampling settings were used without enhanced sampling options. For each compound, the best-ranked binding pose was selected based on GlideScore together with visual inspection of ligand orientation and active-site complementarity.

Binding-mode analysis focused on occupation of the canonical S1 and S4 recognition regions of FXa and on interactions with residues known to contribute to direct inhibitor recognition, particularly Tyr99, Asp189, Phe174, Trp215, and Gly216. Hydrogen bonds, hydrophobic and aromatic contacts, and water-mediated interactions were evaluated using Maestro visualization and interaction-analysis tools. No alternative or allosteric binding sites were explored.

### 4.11. Molecular Dynamics Simulations

The docked complexes of FXa with the ten selected PAX analogs, the experimental-series reference compounds **6y** and **7f**, and the crystallographic reference inhibitor apixaban were subjected to molecular dynamics (MD) simulations using the Desmond module implemented in Schrödinger Maestro 2023-3 (Schrödinger, LLC, New York, NY, USA).

Each protein–ligand complex was solvated in an orthorhombic simulation box containing TIP3P water molecules, with a minimum buffer distance of 10 Å between the protein surface and the simulation-box boundary. Counterions were added to neutralize the systems, and a physiological ionic strength of 0.15 M NaCl was established.

Before the production phase, all systems were relaxed using the default Desmond relaxation protocol, including energy minimization and gradual equilibration. Production simulations were performed under isothermal-isobaric (NPT) conditions at 300 K and 1 atm. Temperature and pressure were controlled using the Nose–Hoover thermostat and Martyna–Tobias–Klein barostat, respectively. The integration time step was 2.0 fs, and trajectory frames were recorded every 100 ps.

An initial nominal 100 ns production simulation was performed for each of the thirteen FXa–ligand complexes. PAX-M09, PAX-M15, and PAX-M18 were subsequently selected for two additional independent nominal 100 ns simulations per compound, giving three runs for each selected ligand and nineteen trajectories in total (1.9 μs of nominal aggregate production time). Initial screening considered protein and ligand RMSD, retention of active-site contacts, and late changes in the interaction pattern, with docking scores and QSAR applicability-domain status providing complementary context. The replicate subset was selected before the final MM/GBSA results were available.

Additional runs used independently randomized initial velocities with different recorded seeds while retaining the corresponding prepared complex and production settings. The three runs per selected ligand were treated as separate 100 ns trajectories, not as a single continuous 300 ns simulation. No 200 ns production trajectories were included in the present dataset.

#### 4.11.1. Trajectory Analysis

Trajectory-derived data were obtained from the Desmond Simulation Interactions Diagram reports and their numerical exports. Protein backbone RMSD was taken from the Prot_Backbone series. Ligand positional deviation was quantified using Lig_wrt_Protein, corresponding to ligand heavy-atom RMSD after alignment of the complex on the reference protein backbone. Lig_wrt_Ligand was reported separately to describe internal conformational deviation after ligand self-alignment. These two ligand RMSD definitions were not used interchangeably.

For descriptive comparison, RMSD and ligand radius-of-gyration values were summarized over the common nominal 50–100 ns interval (exported frames 500–1000 inclusive; 501 frames per run). Means and sample standard deviations describe time-series variability within that interval. The same interval was applied to all runs and was not assumed to establish equilibration or convergence. Late behavior was additionally inspected over the nominal 80–100 ns interval. Protein Cα RMSF values were taken from the full-trajectory P_RMSF export; they were not recalculated for the 50–100 ns window. The binding-site summary was the arithmetic mean across chain-A Tyr99, Phe174, Asp189, Gln192, Ser195, Trp215, Gly216, and Gly218.

Hydrogen bonds, hydrophobic interactions, π–π and π–cation contacts, ionic contacts, and water bridges were examined using the exported interaction records. Numerical residue occupancies were calculated as the percentage of frames that contained at least one interaction of the specified type with that residue, with each residue counted at most once per frame. Combined hydrophobic/aromatic occupancy was calculated from the union of hydrophobic, π–π, and π–cation records rather than by adding percentages. Supplementary contact histograms instead display mean contact counts per frame across the full trajectory, matching the contact-count convention used in the source reports; such values can exceed 1.

Large ligand RMSD was interpreted alongside contact retention rather than used alone as evidence of dissociation. The presence of exported contacts supports continuing protein association but does not establish a unique bound geometry, exclude brief events between saved frames, or determine binding thermodynamics. Replicate trajectories were summarized separately for structural assessment.

#### 4.11.2. MM/GBSA Binding Free Energy Calculations

Prime MM/GBSA calculations were used to obtain comparative endpoint estimates for the thirteen FXa–ligand systems. The calculation protocol specified the OPLS4 force field and VSGB 2.1 implicit-solvent model. Frames from the final nominal 20 ns of the 100 ns production trajectories were used, and binding estimates were defined as (Equation (7)):

(7) ΔGbind = Gcomplex − (Gprotein + Gligand)

Here, Gcomplex, Gprotein, and Gligand denote the calculated complex, receptor, and ligand energies. For each trajectory, the arithmetic mean and standard deviation (SD) were calculated directly from the individual frame-wise MM/GBSA ΔGbind estimates obtained over the final nominal 20 ns. For PAX-M09, PAX-M15, and PAX-M18, the three independently seeded 100 ns trajectories were analyzed and reported separately rather than pooled. The reported SD therefore describes frame-wise variation within the corresponding trajectory and should not be interpreted as an SD across independent replicate means or as a standard error of the mean. For the remaining compounds, only one trajectory was available. The 80–100 ns sampling window was treated as a terminal analysis interval, not as proof that every complex had reached equilibrium. Accordingly, the estimates describe the sampled end-state ensemble, which may differ from the starting pose. MM/GBSA values were interpreted as approximate comparative descriptors, not as experimentally measured binding free energies, IC_50_ values, or evidence of clinical efficacy.

### 4.12. In Silico ADMET Profiling

The ten compounds selected for the receptor-based stage were subjected to in silico ADMET profiling together with the experimental-series controls **6y** and **7f** and the approved FXa inhibitor rivaroxaban. SwissADME was used to calculate physicochemical properties, consensus lipophilicity, aqueous solubility estimates, gastrointestinal (GI) absorption, blood–brain barrier (BBB) permeation, P-glycoprotein (P-gp) substrate status, cytochrome P450 inhibition, drug-likeness filters, medicinal chemistry alerts, bioavailability score, and synthetic accessibility [13].

pkCSM was used as an independent graph-signature-based ADMET predictor [29]. The extracted endpoints comprised water solubility, Caco-2 permeability, human intestinal absorption, skin permeability, P-gp substrate and inhibitor status, volume of distribution at steady state (VDss), fraction unbound, BBB and CNS permeability, CYP2D6/CYP3A4 substrate status, inhibition of CYP1A2, CYP2C19, CYP2C9, CYP2D6, and CYP3A4, total clearance, renal OCT2 substrate status, AMES mutagenicity, maximum tolerated dose, hERG-I and hERG-II inhibition, acute and chronic rat toxicity estimates, hepatotoxicity, skin sensitization, and ecological toxicity endpoints. SwissADME and pkCSM results were interpreted as model-dependent screening predictions rather than experimental measurements; discordant classifications were explicitly retained and not averaged or forced into a consensus call.

The SwissADME BOILED-Egg representation was also used to visualize the relationships among WLOGP, topological polar surface area (TPSA), predicted intestinal absorption, BBB permeation, and P-gp substrate classification [14].

## 5. Conclusions

This study demonstrates the value of combining a constrained QSAR model with explicit applicability-domain assessment and receptor-based refinement for prospective FXa inhibitor prioritization. The integrated workflow identified PAX-M09, PAX-M15, and PAX-M18 as the most relevant candidates for further investigation, while also showing that ligand-based predictions, docking, MD, and MM/GBSA provide complementary rather than interchangeable information. The results should therefore be regarded as a computational prioritization framework rather than evidence of experimentally confirmed potency. Future work should focus on synthesis, biochemical FXa inhibition and selectivity testing, direct binding characterization where appropriate, and experimental ADME evaluation, particularly in view of the predicted solubility and metabolic liabilities.

## Figures and Tables

**Figure 1:**
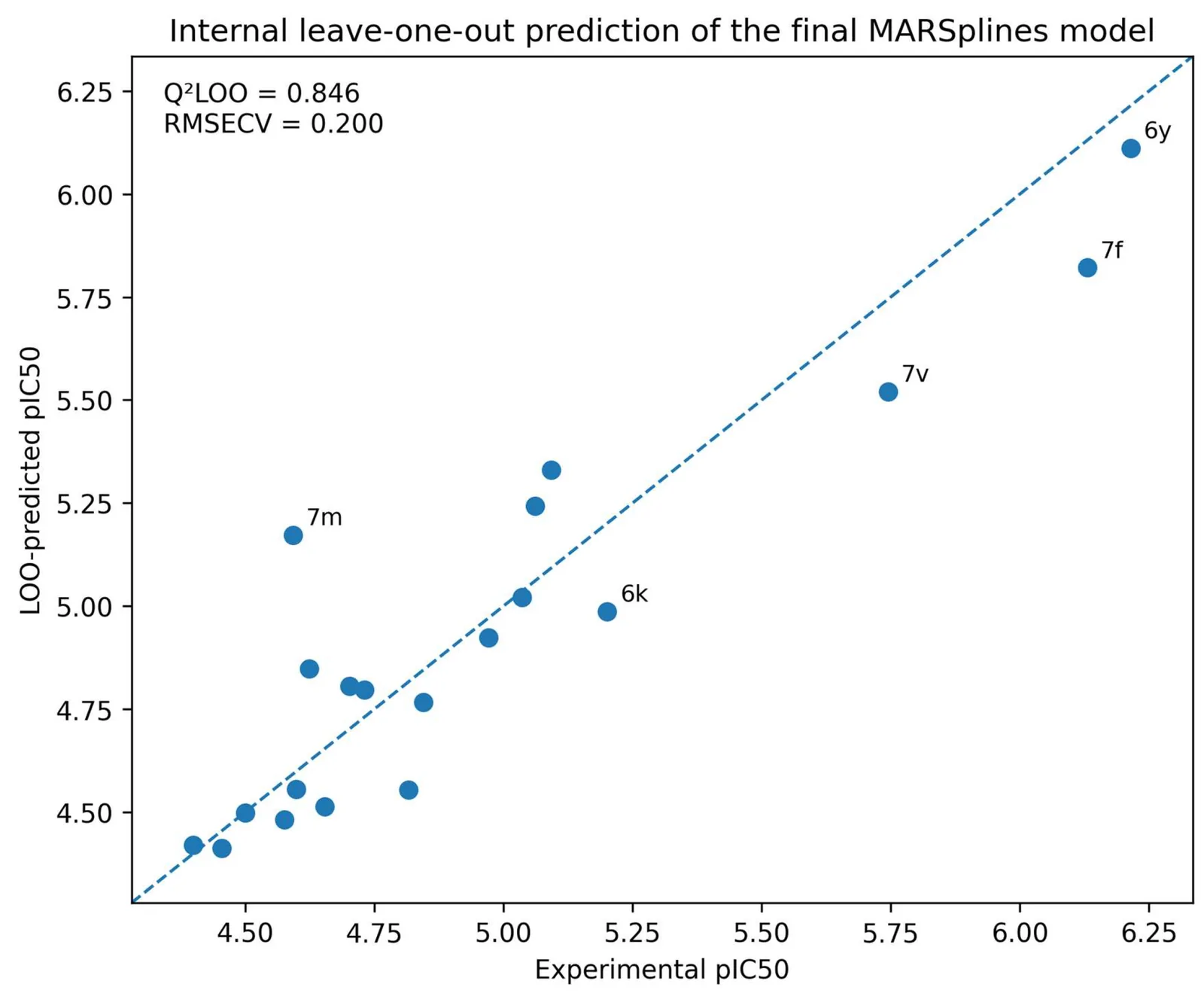
Experimental versus leave-one-out-predicted pIC_50_ values for the final constrained additive MARSplines model. The dashed line represents the ideal y = x relationship. Only selected compounds are labeled to preserve readability.

**Figure 2:**
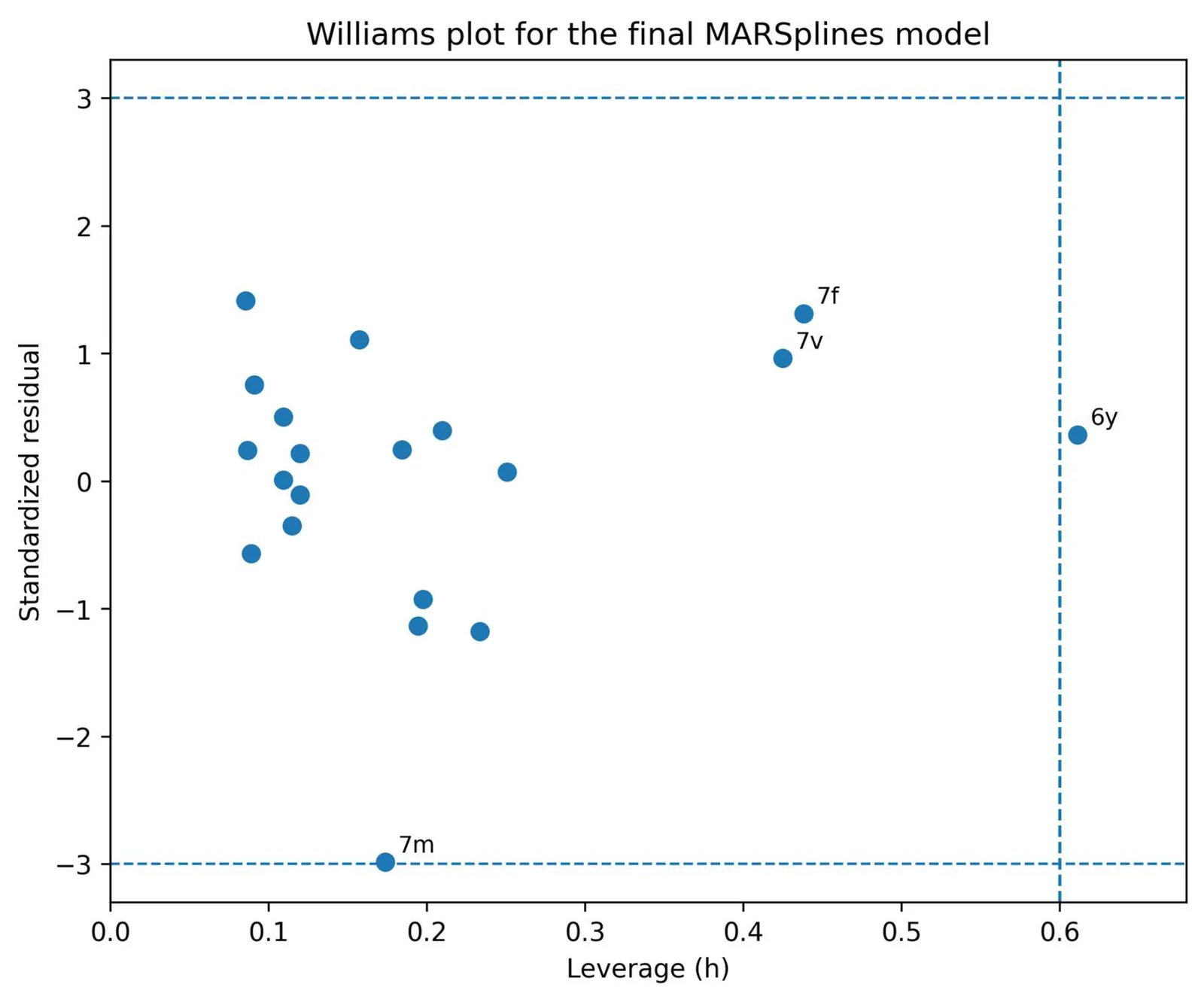
Williams plot for the final MARSplines model. The vertical dashed line indicates the warning leverage h* = 0.60, and the horizontal dashed lines indicate standardized residual limits of ±3. Compound **6y** lies slightly above the leverage threshold, whereas no response outlier exceeds the residual limits.

**Figure 3:**
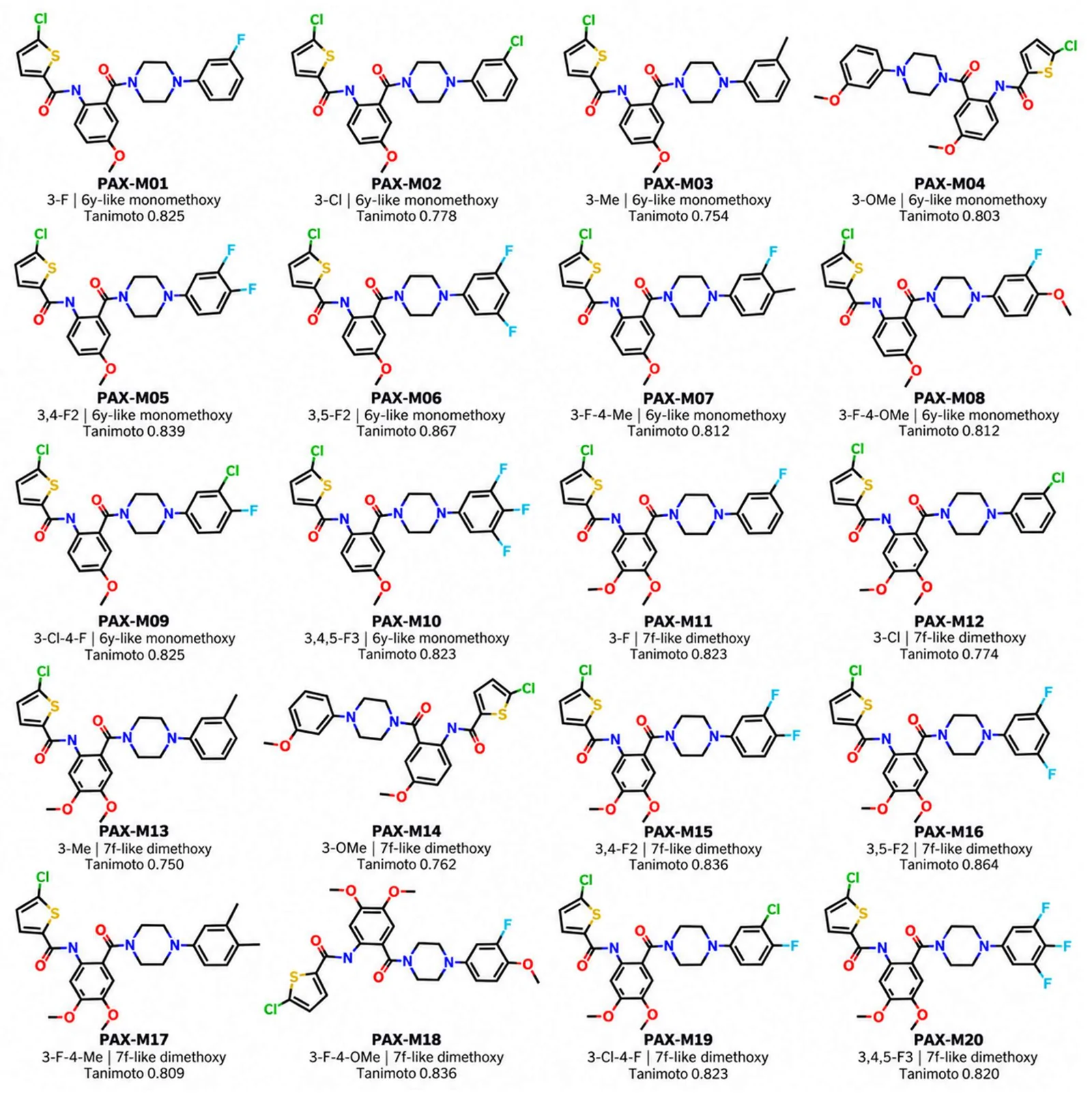
Structure grid of the twenty newly designed PAX-M01-PAX-M20 analogs. The compounds were generated as matched monomethoxy/dimethoxy pairs and subsequently subjected to DFT optimization, alvaDesc descriptor calculation, prospective MARS prediction, and applicability-domain analysis.

**Figure 4:**
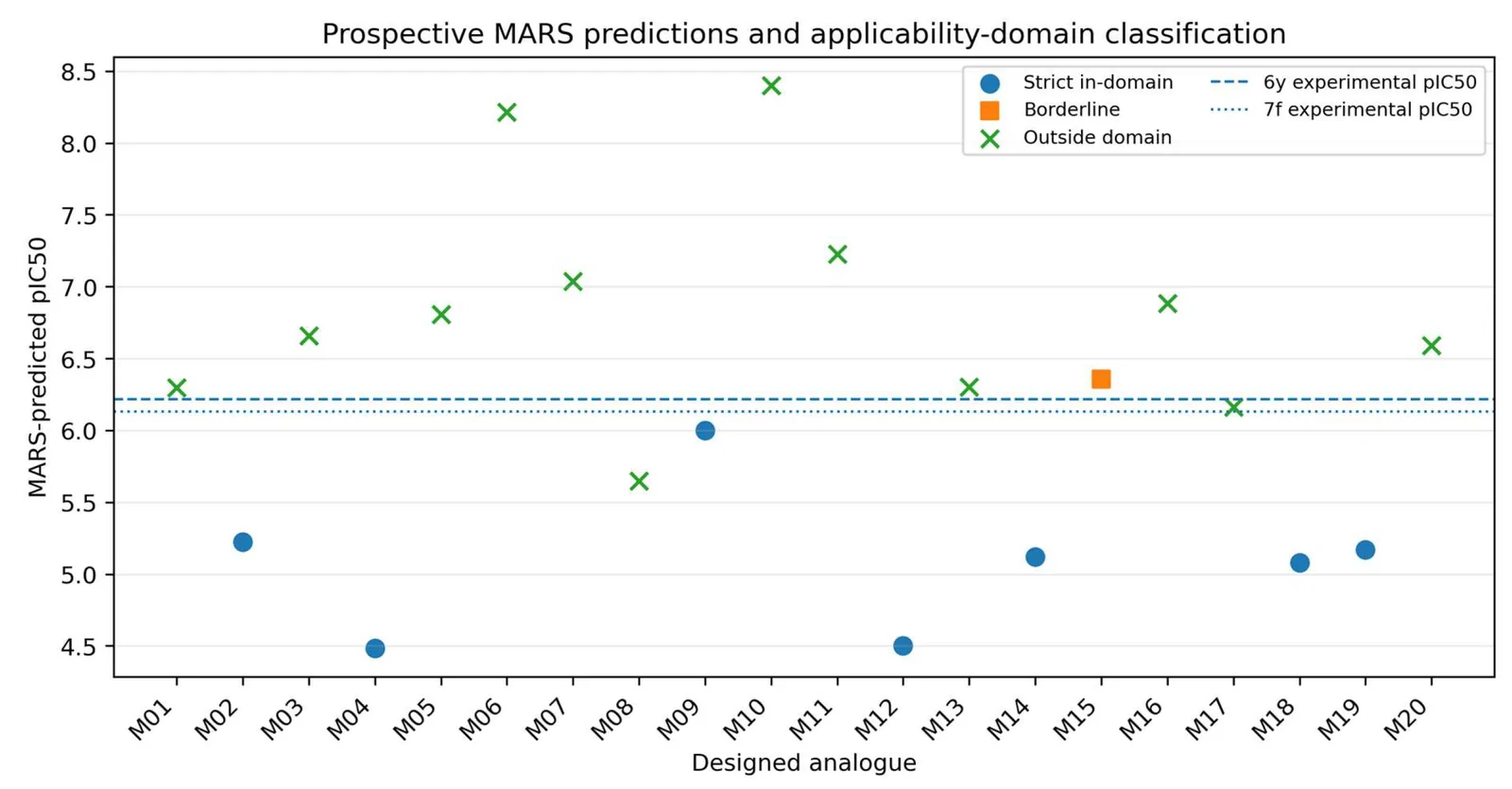
Prospective MARS-predicted pIC_50_ values for the twenty designed analogs, classified as strict in-domain, borderline, or outside-domain. Horizontal reference lines indicate the experimental pIC_50_ values of **6y** and **7f**. Outside-domain raw predictions are displayed for transparency but are not interpreted quantitatively.

**Figure 5:**
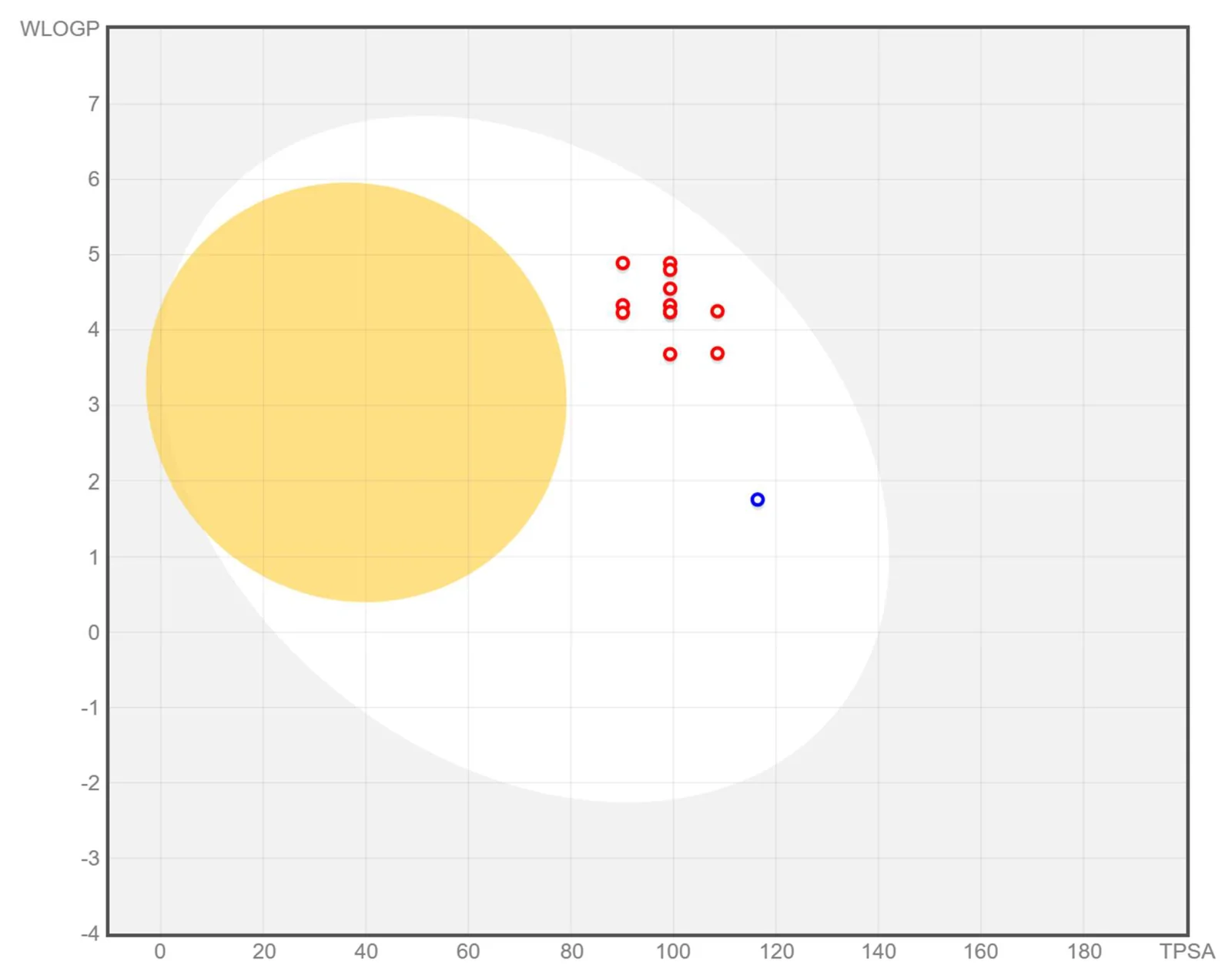
BOILED-Egg plot generated from SwissADME for the ten selected PAX analogs, **6y**, **7f**, and rivaroxaban [13,14]. The white region denotes the high-probability human intestinal absorption (HIA) space, and the yellow region denotes the BBB-permeant space. Red circles correspond to SwissADME-predicted P-gp non-substrates (all PAX compounds plus **6y** and **7f**), whereas the blue circle corresponds to rivaroxaban, predicted as a P-gp substrate.

**Figure 6:**
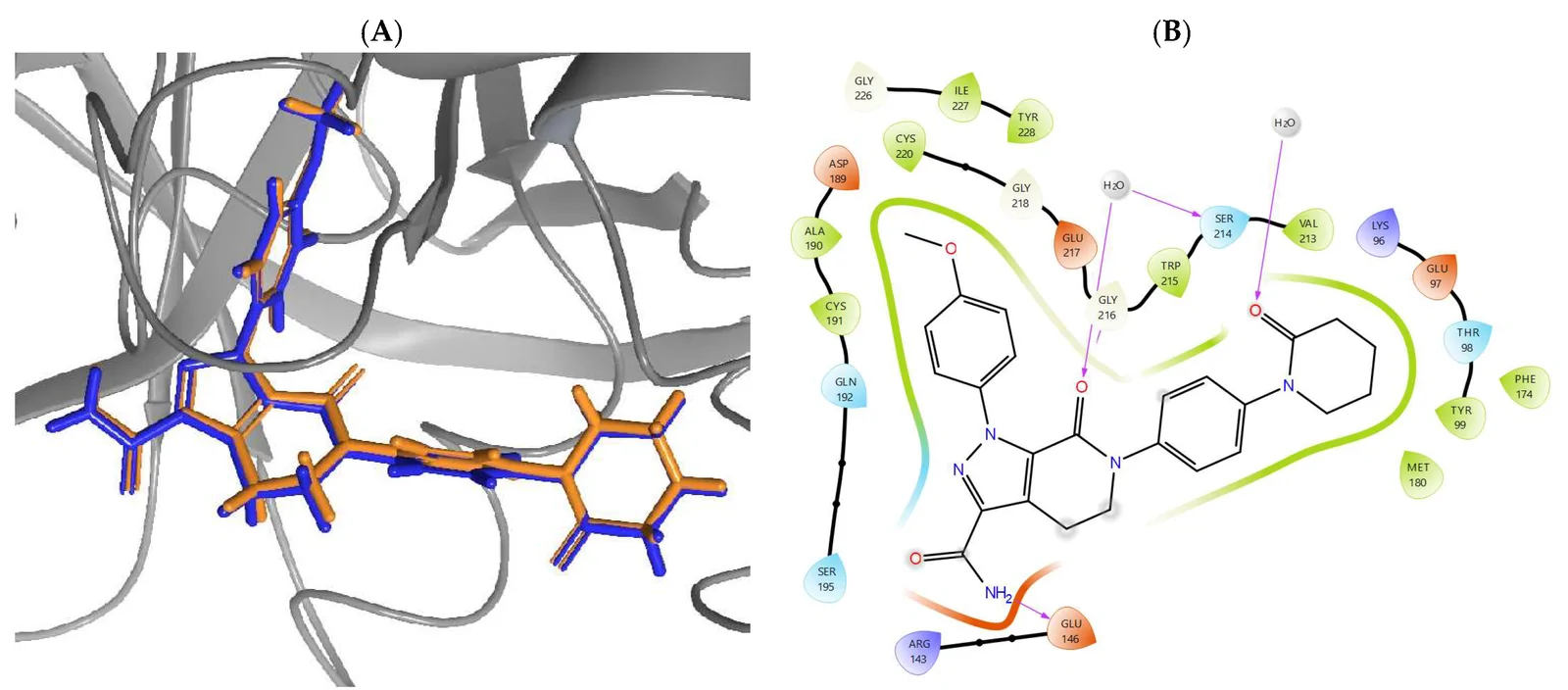
Apixaban reference complex in human factor Xa (FXa; PDB ID: 2P16). (**A**) Superposition of the crystallographic (blue) and redocked (orange) apixaban conformations within the FXa binding site. The protein is shown as a gray cartoon. The redocking RMSD was 0.953 Å. (**B**) Two-dimensional interaction diagram of the selected docked FXa–apixaban complex.

**Figure 7:**
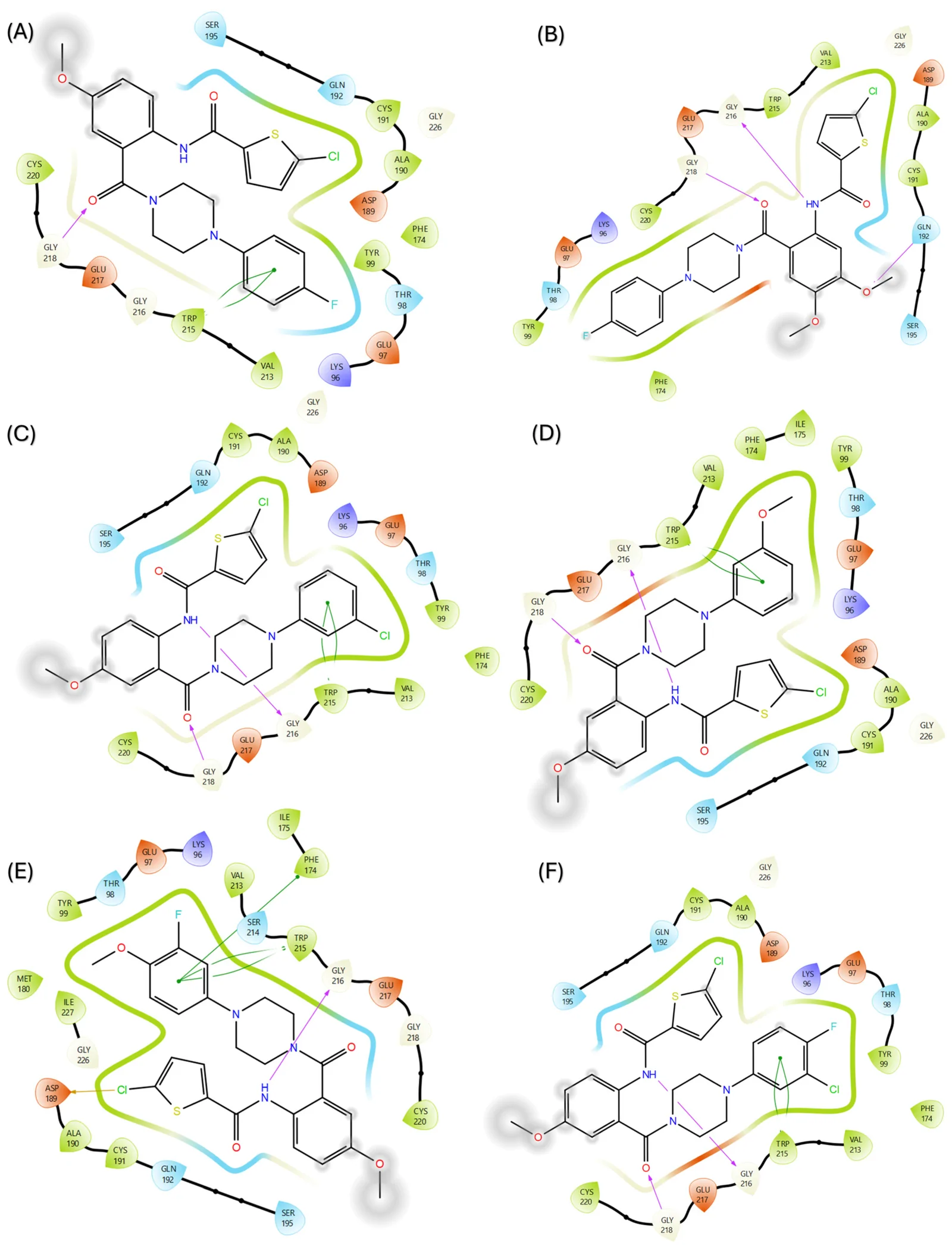
Two-dimensional interaction diagrams of the selected docked FXa-ligand complexes. (**A**) **6y**, (**B**) **7f**, (**C**) PAX-M02, (**D**) PAX-M04, (**E**) PAX-M08, and (**F**) PAX-M09.

**Figure 8:**
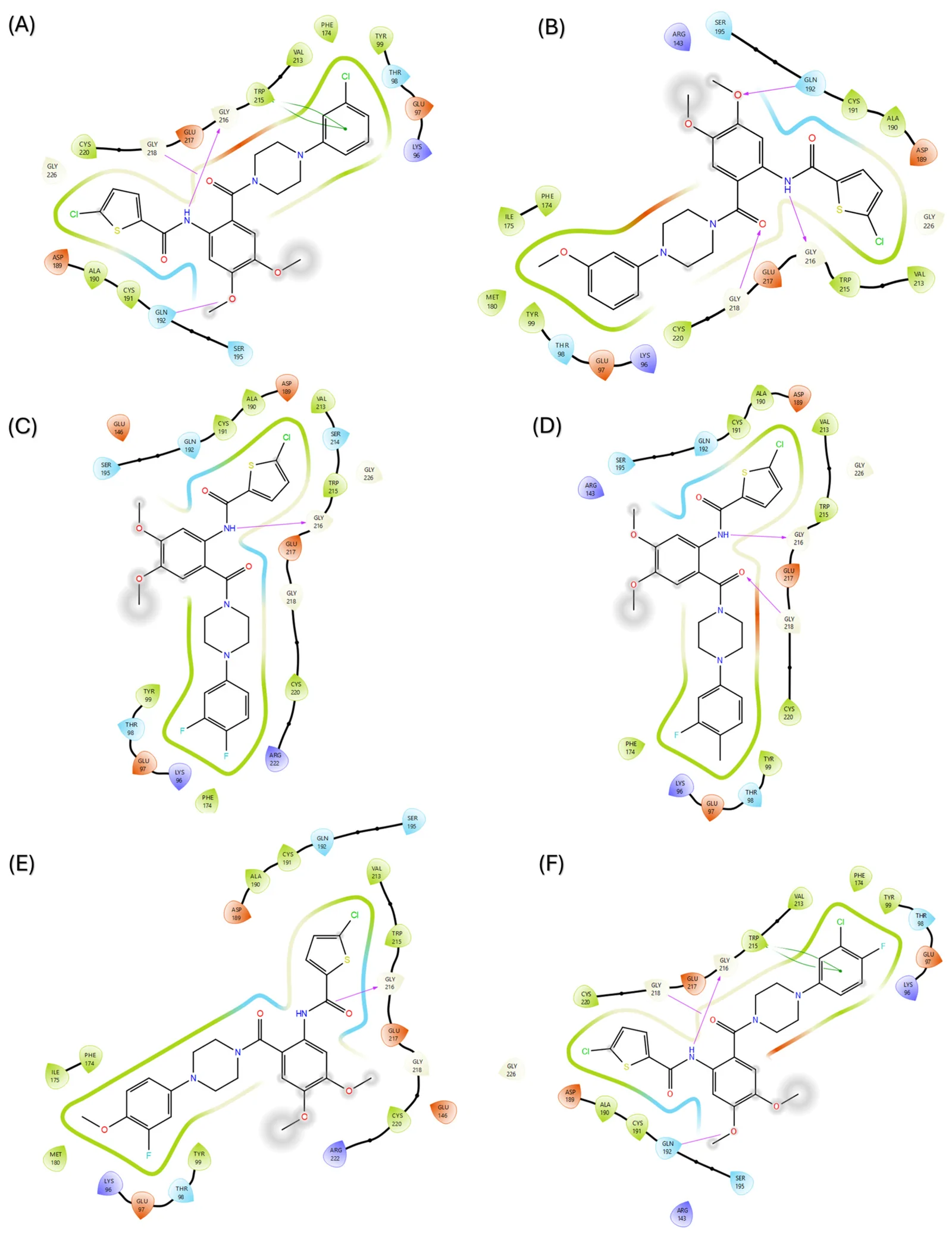
Two-dimensional interaction diagrams of the selected docked FXa-ligand complexes. (**A**) PAX-M12, (**B**) PAX-M14, (**C**) PAX-M15, (**D**) PAX-M17, (**E**) PAX-M18, and (**F**) PAX-M19.

**Figure 9:**
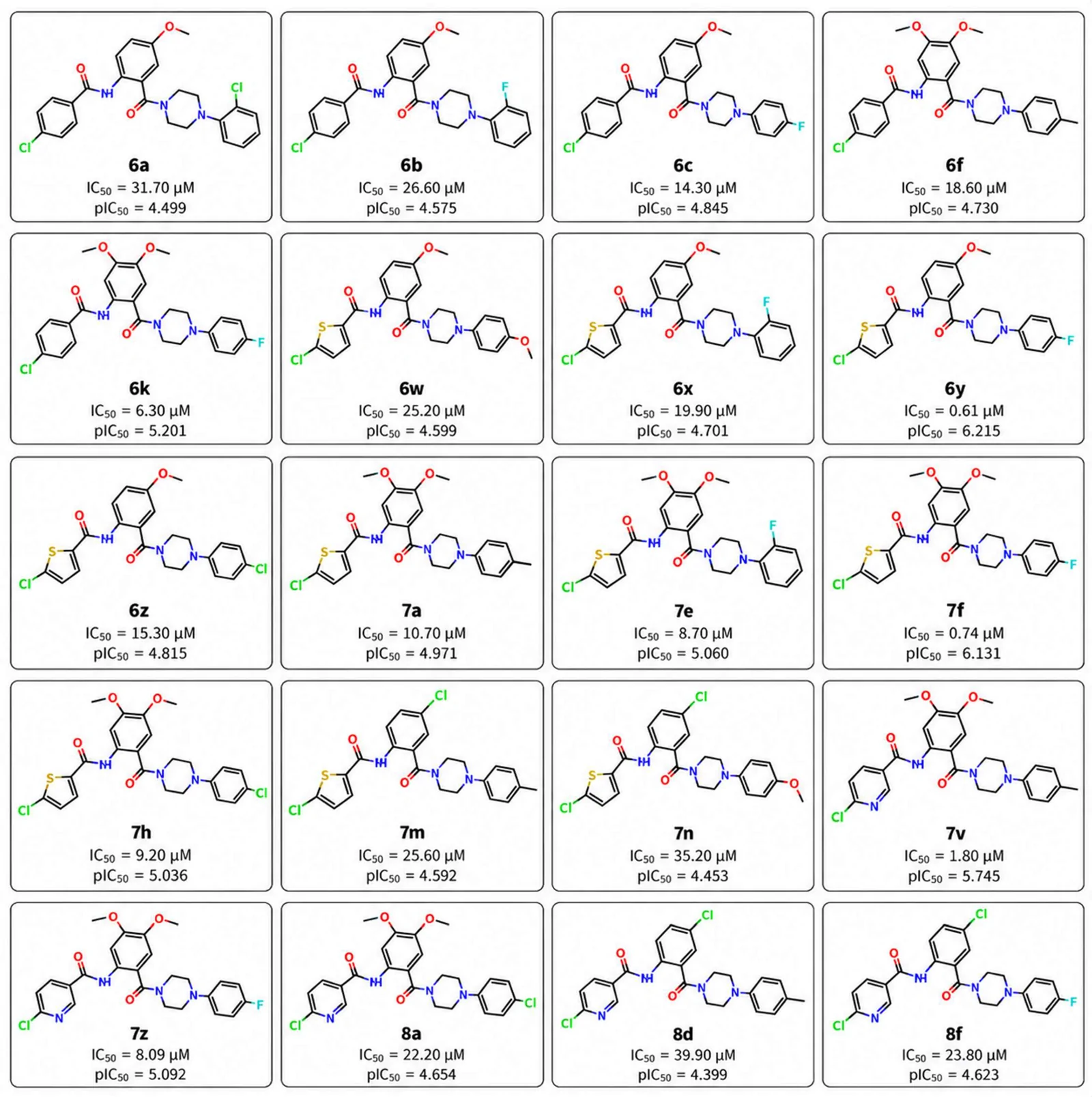
Structures of the twenty literature-derived training compounds used for QSAR modeling. Experimental IC_50_ and calculated pIC_50_ values are shown below each structure. Structures were redrawn from the source series [4].

**Table 1:** Comparative performance of the candidate QSAR approaches. For MLR and MARSplines, statistics refer to the selected fixed model specification. Hyperparameter-dependent alternative algorithms were evaluated using nested internal leave-one-out procedures. Values are used for model comparison and do not represent external validation.

Model	Terms	R^2^	Adj. R^2^	Q2_LOO	RMSECV	MAE_LOO
Additive MARSplines	3	0.904	0.886	0.846	0.200	0.149
MLR 3 descriptors	3	0.668	0.606	0.533	0.349	0.259
MLR 4 descriptors	4	0.741	0.672	0.544	0.345	0.290
PLS (nested LOO)				0.427	0.386	0.296
Ridge (nested LOO)				0.327	0.419	0.297
Elastic Net (nested LOO)				−0.013	0.514	0.403
SVR RBF (nested LOO)				0.191	0.459	0.323
SVR linear (nested LOO)				−0.088	0.532	0.418

**Table 2:** Basis functions of the final constrained additive MARSplines model.

Basis Function	Descriptor	Definition	Coefficient	Active Support (n)
BF1	Mor21m	max(0, Mor21m + 0.95363491)	+5.637205	7
BF2	RDF055e	max(0, RDF055e − 16.71696820)	+0.162580	14
BF3	SHED_AL	max(0, 11.37792108 − SHED_AL)	+0.597256	10

**Table 3:** Statistical and internal-validation parameters of the final additive MARSplines model.

Parameter	Value	Parameter	Value
Number of compounds	20	R^2^	0.9041
Active descriptors/basis functions	3/3	Adjusted R^2^	0.8861
RMSEC	0.1581	Calibration MAE	0.1161
Q2_LOO	0.8457	RMSECV_LOO	0.2005
MAE_LOO	0.1494	Repeated 5-fold Q2	0.8298 ± 0.0407
Repeated 5-fold RMSE	0.2093	Repeated 5-fold MAE	0.1558
Maximum VIF	1.103	F statistic	50.26
Y-randomization permutations	1000	Randomized Q2 ≥ observed	0
Mean randomized Q2	−0.3774	Maximum randomized Q2	0.6030

**Table 4:** Twenty newly designed piperazinylanthranilamide analogs were generated as matched monomethoxy/dimethoxy P4-substitution pairs. Structural definitions and nearest-source Morgan similarity are shown; all compounds subsequently completed the prospective DFT/alvaDesc workflow.

Code	Core	P4 Substitution	Formula	Nearest Source Ref.	Tanimoto
PAX-M01	monomethoxy	3-F	C23H21ClFN3O3S	6y	0.825
PAX-M02	monomethoxy	3-Cl	C23H21Cl2N3O3S	6y	0.778
PAX-M03	monomethoxy	3-Me	C24H24ClN3O3S	6y	0.754
PAX-M04	monomethoxy	3-OMe	C24H24ClN3O4S	6y	0.803
PAX-M05	monomethoxy	3,4-F2	C23H20ClF2N3O3S	6y	0.839
PAX-M06	monomethoxy	3,5-F2	C23H20ClF2N3O3S	6y	0.867
PAX-M07	monomethoxy	3-F-4-Me	C24H23ClFN3O3S	6y	0.812
PAX-M08	monomethoxy	3-F-4-OMe	C24H23ClFN3O4S	6y	0.812
PAX-M09	monomethoxy	3-Cl-4-F	C23H20Cl2FN3O3S	6y	0.825
PAX-M10	monomethoxy	3,4,5-F3	C23H19ClF3N3O3S	6y	0.823
PAX-M11	dimethoxy	3-F	C24H23ClFN3O4S	7f	0.823
PAX-M12	dimethoxy	3-Cl	C24H23Cl2N3O4S	7f	0.774
PAX-M13	dimethoxy	3-Me	C25H26ClN3O4S	7f	0.750
PAX-M14	dimethoxy	3-OMe	C25H26ClN3O5S	7f	0.762
PAX-M15	dimethoxy	3,4-F2	C24H22ClF2N3O4S	7f	0.836
PAX-M16	dimethoxy	3,5-F2	C24H22ClF2N3O4S	7f	0.864
PAX-M17	dimethoxy	3-F-4-Me	C25H25ClFN3O4S	7f	0.809
PAX-M18	dimethoxy	3-F-4-OMe	C25H25ClFN3O5S	7f	0.836
PAX-M19	dimethoxy	3-Cl-4-F	C24H22Cl2FN3O4S	7f	0.823
PAX-M20	dimethoxy	3,4,5-F3	C24H21ClF3N3O4S	7f	0.820

**Table 5:** Prospective MARSplines predictions and applicability-domain classification for PAX-M01-PAX-M20. Raw pIC_50_ values for outside-domain compounds are shown for transparency but should not be interpreted quantitatively.

Code	Core	P4	Pred. pIC_50_	Pred. IC_50_ (µM)	Leverage	AD
PAX-M01	mono-OMe	3-F	6.298	0.504	1.300	Outside
PAX-M02	mono-OMe	3-Cl	5.224	5.970	0.296	In domain
PAX-M03	mono-OMe	3-Me	6.658	0.220	1.687	Outside
PAX-M04	mono-OMe	3-OMe	4.483	32.916	0.100	In domain
PAX-M05	mono-OMe	3,4-F2	6.807	0.156	1.430	Outside
PAX-M06	mono-OMe	3,5-F2	8.217	0.006	6.807	Outside
PAX-M07	mono-OMe	3-F-4-Me	7.038	0.092	4.736	Outside
PAX-M08	mono-OMe	3-F-4-OMe	5.646	2.258	0.670	Outside
PAX-M09	mono-OMe	3-Cl-4-F	6.001	0.997	0.311	In domain
PAX-M10	mono-OMe	3,4,5-F3	8.402	0.004	3.928	Outside
PAX-M11	di-OMe	3-F	7.226	0.059	4.643	Outside
PAX-M12	di-OMe	3-Cl	4.500	31.607	0.109	In domain
PAX-M13	di-OMe	3-Me	6.301	0.500	1.322	Outside
PAX-M14	di-OMe	3-OMe	5.119	7.601	0.417	In domain
PAX-M15	di-OMe	3,4-F2	6.356	0.440	0.527	Borderline
PAX-M16	di-OMe	3,5-F2	6.883	0.131	1.154	Outside
PAX-M17	di-OMe	3-F-4-Me	6.164	0.686	0.838	Outside
PAX-M18	di-OMe	3-F-4-OMe	5.081	8.294	0.298	In domain
PAX-M19	di-OMe	3-Cl-4-F	5.169	6.772	0.169	In domain
PAX-M20	di-OMe	3,4,5-F3	6.591	0.256	1.130	Outside

**Table 6:** Ten designed analogs were selected for the receptor-based stage following QSAR prediction and applicability domain assessment. Priority tiers and selection rationale are described in the accompanying text.

Tier	Code	Core	P4	Pred. pIC_50_	Leverage
Primary	PAX-M09	mono-OMe	3-Cl-4-F	6.001	0.311
Primary	PAX-M02	mono-OMe	3-Cl	5.224	0.296
Primary	PAX-M19	di-OMe	3-Cl-4-F	5.169	0.169
Primary	PAX-M14	di-OMe	3-OMe	5.119	0.417
Primary	PAX-M18	di-OMe	3-F-4-OMe	5.081	0.298
Primary	PAX-M12	di-OMe	3-Cl	4.500	0.109
Primary	PAX-M04	mono-OMe	3-OMe	4.483	0.100
Borderline	PAX-M15	di-OMe	3,4-F2	6.356	0.527
Exploratory	PAX-M17	di-OMe	3-F-4-Me	6.164	0.838
Exploratory	PAX-M08	mono-OMe	3-F-4-OMe	5.646	0.670

**Table 7:** Selected SwissADME and pkCSM absorption/distribution descriptors for the ten prioritized PAX analogs, source-series controls, and rivaroxaban.

Compound	Swiss cLogP	Swiss ESOL LogS	pkCSM LogS	Caco-2	HIA (%)	logBB	logPS	P-gp Swiss/pkCSM
PAX-M09	4.83	−6.72	−5.355	1.004	89.4	−0.409	−1.851	No/Yes
PAX-M02	4.60	−6.55	−5.673	1.002	89.4	−0.372	−1.805	No/Yes
PAX-M19	4.89	−6.80	−5.361	1.027	90.2	−0.782	−2.021	No/Yes
PAX-M14	3.92	−6.11	−4.751	1.099	94.5	−0.334	−2.287	No/Yes
PAX-M18	4.26	−6.28	−4.749	1.155	93.6	−0.504	−3.005	No/Yes
PAX-M12	4.62	−6.63	−5.772	1.025	90.2	−0.591	−1.975	No/Yes
PAX-M04	4.13	−6.03	−4.629	1.022	94.2	−0.290	−2.131	No/Yes
PAX-M15	4.64	−6.36	−5.140	1.006	91.0	−0.814	−2.887	No/Yes
PAX-M17	4.62	−6.50	−5.556	1.004	91.6	−0.606	−2.057	No/Yes
PAX-M08	4.26	−6.20	−5.134	1.029	91.3	−0.658	−2.129	No/Yes
6y	4.27	−6.12	−5.191	0.949	89.7	−0.389	−1.956	No/Yes
7f	4.17	−6.20	−5.279	0.972	90.5	−0.641	−2.127	No/Yes
Rivaroxaban	2.25	−4.00	−4.973	1.109	93.8	−0.934	−2.596	Yes/Yes

cLogP, SwissADME consensus LogP; HIA, pkCSM human intestinal absorption. SwissADME predicted High GI absorption and BBB permeant = No for all 13 molecules. P-gp values are reported as SwissADME/pkCSM substrate calls; the PAX series and **6y**/**7f** were classified as non-substrates by SwissADME but as substrates by pkCSM.

**Table 8:** Selected pkCSM excretion and toxicity outputs for the ten prioritized PAX analogs, source-series controls, and rivaroxaban.

Compound	Clearance	MTD	LD50	LOAEL	AMES	hERG I/II	Hepatotoxicity
PAX-M09	0.347	0.082	2.315	1.324	No	No/Yes	Yes
PAX-M02	0.308	0.023	2.259	1.323	No	No/Yes	Yes
PAX-M19	0.603	0.111	2.355	1.448	No	No/Yes	Yes
PAX-M14	0.790	−0.146	2.319	1.694	No	No/Yes	Yes
PAX-M18	0.739	−0.097	2.269	1.600	No	No/Yes	Yes
PAX-M12	0.564	0.053	2.334	1.460	No	No/Yes	No
PAX-M04	0.537	−0.122	2.361	1.111	No	No/Yes	Yes
PAX-M15	0.558	0.157	2.332	1.714	No	No/Yes	Yes
PAX-M17	0.498	0.081	2.393	1.516	No	No/Yes	Yes
PAX-M08	0.483	0.059	2.289	1.299	No	No/Yes	Yes
6y	0.231	0.114	2.230	1.365	No	No/Yes	Yes
7f	0.487	0.139	2.295	1.459	No	No/Yes	Yes
Rivaroxaban	0.298	−0.439	2.907	1.963	No	No/Yes	Yes

MTD, pkCSM maximum tolerated dose model output; LD50, oral rat acute toxicity model output; LOAEL, oral rat chronic toxicity model output. All compounds were predicted as hERG-I non-inhibitors and hERG-II inhibitors.

**Table 9:** Docking scores and trajectory-based MM/GBSA estimates for the thirteen FXa–ligand complexes.

Compound	Glide Score	MM/GBSA ΔGbind ± SD [kcal/mol]		
		R1	R2	R3
Apixaban	−11.362	−77.31 ± 3.42	—	—
6y	−10.086	−54.11 ± 5.03	—	—
7f	−9.331	−50.85 ± 4.87	—	—
PAX-M02	−9.621	−49.64 ± 4.83	—	—
PAX-M04	−9.039	−48.34 ± 5.62	—	—
PAX-M08	−9.554	−52.71 ± 8.23	—	—
PAX-M09	−9.952	−62.41 ± 3.86	−64.86 ± 4.42	−62.06 ± 3.64
PAX-M12	−9.910	−54.81 ± 5.01	—	—
PAX-M14	−9.654	−54.65 ± 3.89	—	—
PAX-M15	−9.565	−68.76 ± 3.21	−70.11 ± 3.76	−69.42 ± 3.14
PAX-M17	−10.267	−42.85 ± 3.06	—	—
PAX-M18	−9.824	−60.13 ± 5.43	−62.48 ± 4.83	−61.95 ± 5.32
PAX-M19	−10.223	−48.73 ± 6.42	—	—

MM/GBSA values are arithmetic means ± SD of frame-wise estimates from the final nominal 20 ns of each trajectory. R1 denotes the initial trajectory; R2 and R3 denote the two additional independently seeded trajectories for PAX-M09, PAX-M15, and PAX-M18. Replicates were analyzed separately and were not pooled. SD describes frame-wise dispersion within each trajectory. Glide and MM/GBSA scales are not interchangeable, and MM/GBSA estimates should not be interpreted as experimental binding free energies.

**Table 10:** Initial-run structural descriptors for the thirteen FXa complexes.

Complex	Backbone RMSD (Å)	Ligand RMSD Protein-Aligned (Å)	Ligand RMSD Self-Aligned (Å)	Mean Site Cα RMSF (Å)
Apixaban	1.38 ± 0.10	0.90 ± 0.20	0.43 ± 0.10	0.56
6y	1.35 ± 0.10	4.65 ± 0.23	1.56 ± 0.12	0.71
7f	1.23 ± 0.09	6.07 ± 0.42	1.99 ± 0.42	0.70
PAX-M02	1.49 ± 0.12	6.07 ± 0.21	1.32 ± 0.09	0.64
PAX-M04	1.46 ± 0.15	2.11 ± 0.39	0.83 ± 0.12	0.69
PAX-M08	1.46 ± 0.10	2.72 ± 0.49	1.34 ± 0.14	0.76
PAX-M09	1.39 ± 0.11	2.18 ± 0.39	0.82 ± 0.10	0.66
PAX-M12	1.27 ± 0.12	2.91 ± 1.07	1.24 ± 0.36	0.60
PAX-M14	1.40 ± 0.12	5.46 ± 0.22	1.38 ± 0.12	0.76
PAX-M15	1.37 ± 0.10	1.59 ± 0.33	1.20 ± 0.11	0.60
PAX-M17	1.36 ± 0.13	4.80 ± 0.45	1.37 ± 0.12	0.70
PAX-M18	1.19 ± 0.09	2.38 ± 0.28	1.27 ± 0.12	0.76
PAX-M19	1.60 ± 0.13	5.94 ± 0.63	1.54 ± 0.32	0.84

RMSD values are mean ± sample SD over the common nominal 50–100 ns interval, separately for each initial run. RMSF values are full-trajectory estimates averaged over the eight chain-A residues defined in Section 4.11.1; no SD across residues is presented as uncertainty.

**Table 11:** Run-resolved structural descriptors for apixaban and the three replicated PAX complexes.

Complex	Run	Backbone RMSD (Å)	Ligand RMSD Protein-Aligned (Å)	Ligand RMSD Self-Aligned (Å)	Ligand rGyr (Å)
Apixaban	R1	1.38 ± 0.10	0.90 ± 0.20	0.43 ± 0.10	5.09 ± 0.04
PAX-M09	R1	1.39 ± 0.11	2.18 ± 0.39	0.82 ± 0.10	5.26 ± 0.08
PAX-M09	R2	1.48 ± 0.12	5.79 ± 0.20	1.42 ± 0.11	5.49 ± 0.07
PAX-M09	R3	1.30 ± 0.10	2.65 ± 0.38	0.85 ± 0.09	5.25 ± 0.07
PAX-M15	R1	1.37 ± 0.10	1.59 ± 0.33	1.20 ± 0.11	5.11 ± 0.07
PAX-M15	R2	1.35 ± 0.11	2.11 ± 0.50	1.27 ± 0.36	5.17 ± 0.14
PAX-M15	R3	1.30 ± 0.09	1.78 ± 0.43	1.02 ± 0.29	5.21 ± 0.10
PAX-M18	R1	1.19 ± 0.09	2.38 ± 0.28	1.27 ± 0.12	5.25 ± 0.09
PAX-M18	R2	1.27 ± 0.09	3.50 ± 0.55	1.61 ± 0.29	5.55 ± 0.18
PAX-M18	R3	1.60 ± 0.10	3.09 ± 0.56	1.23 ± 0.17	5.21 ± 0.07

Means ± sample SD describe fluctuations over nominal 50–100 ns within each run. R1 denotes the initial screen and R2/R3 the additional independently seeded runs. The table does not average away the distinct second-run behavior of M09. Ligand rGyr is not the protein radius of gyration. Apixaban was simulated once. These structural replicate values complement the run-resolved MM/GBSA results reported in Table 9.

**Table 12:** Literature-derived piperazinylanthranilamide dataset used for QSAR model development. IC_50_ values are mean ± S.E.M. as reported by Khadse et al. [4]; pIC_50_ values were calculated from the mean IC_50_ values according to Equation (6).

Code	P1 Fragment	X (Core)	Y (P4 Phenyl)	IC_50_ (µM)	pIC_50_
6a	4-chlorophenyl	5-OMe	2-Cl	31.70 ± 3.20	4.499
6b	4-chlorophenyl	5-OMe	2-F	26.60 ± 3.70	4.575
6c	4-chlorophenyl	5-OMe	4-F	14.30 ± 2.60	4.845
6f	4-chlorophenyl	4,5-(OMe)2	4-Me	18.60 ± 3.20	4.730
6k	4-chlorophenyl	4,5-(OMe)2	4-F	6.30 ± 1.40	5.201
6w	5-chloro-2-thienyl	5-OMe	4-OMe	25.20 ± 4.30	4.599
6x	5-chloro-2-thienyl	5-OMe	2-F	19.90 ± 4.50	4.701
6y	5-chloro-2-thienyl	5-OMe	4-F	0.61 ± 0.07	6.215
6z	5-chloro-2-thienyl	5-OMe	4-Cl	15.30 ± 2.70	4.815
7a	5-chloro-2-thienyl	4,5-(OMe)2	4-Me	10.70 ± 1.60	4.971
7e	5-chloro-2-thienyl	4,5-(OMe)2	2-F	8.70 ± 1.30	5.060
7f	5-chloro-2-thienyl	4,5-(OMe)2	4-F	0.74 ± 0.14	6.131
7h	5-chloro-2-thienyl	4,5-(OMe)2	4-Cl	9.20 ± 2.10	5.036
7m	5-chloro-2-thienyl	5-Cl	4-Me	25.60 ± 6.30	4.592
7n	5-chloro-2-thienyl	5-Cl	4-OMe	35.20 ± 5.80	4.453
7v	6-chloro-3-pyridyl	4,5-(OMe)2	4-Me	1.80 ± 0.30	5.745
7z	6-chloro-3-pyridyl	4,5-(OMe)2	4-F	8.09 ± 1.60	5.092
8a	6-chloro-3-pyridyl	4,5-(OMe)2	4-Cl	22.20 ± 4.10	4.654
8d	6-chloro-3-pyridyl	5-Cl	4-Me	39.90 ± 8.40	4.399
8f	6-chloro-3-pyridyl	5-Cl	4-F	23.80 ± 4.70	4.623

Rivaroxaban was used as the reference inhibitor in the source study, but was not included in QSAR model development.

## Data Availability

The original contributions presented in this study are included in the article. Further inquiries can be directed to the corresponding author. The experimental FXa inhibition data analyzed in this study were reported previously by Khadse et al. [4].

## Supplementary Materials

The following supporting information can be downloaded at https://www.mdpi.com/article/10.3390/ijms27209204/s1.

## Abbreviations

The following abbreviations are used in this manuscript:

Abbreviation	Definition
3D-MoRSE	Three-dimensional molecule representation of structures based on electron diffraction
AD	Applicability domain
ADME	Absorption, distribution, metabolism, and excretion
ADMET	Absorption, distribution, metabolism, excretion, and toxicity
AMES	Ames mutagenicity test (pkCSM endpoint label)
B3LYP	Becke three-parameter hybrid functional with Lee–Yang–Parr correlation
BBB	Blood–brain barrier
BF	Basis function
BOILED-Egg	Brain Or IntestinaL EstimateD permeation model
Cα	Alpha-carbon atom
cLogP	Consensus predicted logP reported by SwissADME
CNS	Central nervous system
CYP	Cytochrome P450
DFT	Density functional theory
ESOL	Model for estimating aqueous solubility from molecular structure
ETKDGv3	Experimental torsion-knowledge distance geometry, version 3
FXa	Coagulation factor Xa
GI	Gastrointestinal
hERG	Human ether-a-go-go-related gene potassium channel
HIA	Human intestinal absorption
IC_50_	Half-maximal inhibitory concentration
LD_50_	Median lethal dose
LOAEL	Lowest-observed-adverse-effect level
logBB	Base-10 logarithm of the brain-to-blood concentration ratio
LogP	Base-10 logarithm of the n-octanol/water partition coefficient
logPS	Base-10 logarithm of the blood–brain permeability–surface area product
LogS	Base-10 logarithm of aqueous solubility expressed in mol/L
LOO	Leave-one-out cross-validation
MAE	Mean absolute error
MARS	Multivariate adaptive regression splines
MD	Molecular dynamics
MLR	Multiple linear regression
MM/GBSA	Molecular mechanics/generalized Born surface area
MMFF94s	Merck molecular force field 94, static variant
MTD	Maximum tolerated dose
MW	Molecular weight
NPT	Isothermal–isobaric ensemble (constant number of particles, pressure, and temperature)
OCT2	Organic cation transporter 2
OPLS4	Optimized potentials for liquid simulations, version 4
P-gp	P-glycoprotein
PAINS	Pan-assay interference compounds
PDB	Protein Data Bank
pIC_50_	Negative base-10 logarithm of IC_50_ expressed in mol/L
PLS	Partial least-squares regression
PRESS	Predicted residual sum of squares
QSAR	Quantitative structure–activity relationship
RBF	Radial basis function
RDF	Radial distribution function
RF	Random Forest
rGyr	Ligand radius of gyration
RMSD	Root-mean-square deviation
RMSE	Root-mean-square error
RMSEC	Root-mean-square error of calibration
RMSECV	Root-mean-square error of cross-validation
RMSF	Root-mean-square fluctuation
SAR	Structure–activity relationship
SD	Standard deviation
SDF	Structure-data file
SEM (S.E.M.)	Standard error of the mean
SVR	Support-vector regression
TIP3P	Three-point transferable intermolecular potential water model
TPSA	Topological polar surface area
VDss	Volume of distribution at steady state
VIF	Variance inflation factor
VSGB	Variable-dielectric generalized Born model
WLOGP	Wildman–Crippen atom-contribution estimate of logP
XP	Extra precision (Glide docking mode)
