# Supplementary material for: QSAR-Guided In Silico Design of Piperazinylanthranilamide Factor Xa Inhibitors: Applicability-Domain Assessment, ADMET Profiling, Molecular Docking, Molecular Dynamics, and MM/GBSA
Source: Int J Mol Sci. 2026 Oct 16;27(20):9204. doi: 10.3390/ijms27209204 (PMC13705391; doi:10.3390/ijms27209204)
Supplement: Supplementary file 1 [file ijms-27-09204-s001.zip › ijms-4626289-supplementary.pdf]

International Journal of
Molecular Sciences

MDPI

Supplementary Materials for

QSAR-Guided In Silico Design of Piperazinylanthranilamide Factor Xa Inhibitors: Applicability-Domain Assessment, ADMET Profiling, Molecular Docking, Molecular Dynamics, and MM/GBSA

Paweł Gordon [1], Michał Janiak [2,3], Marcin Gackowski [2], Monika Zielińska-Pisklak [4] and Łukasz Szeleszczuk [5,*]

[1] University of Health Sciences in Bydgoszcz, Jagiellońska 4 Str., 85-067 Bydgoszcz, Poland; p.gordon@wsnoz.edu.pl
[2] Department of Toxicology and Bromatology, Faculty of Pharmacy, L. Rydygier Collegium Medicum in Bydgoszcz, Nicolaus Copernicus University in Toruń, A. Jurasza 2 Street, 85-089 Bydgoszcz, Poland; michal.janiak@doktorant.umk.pl (M.J.); marcin.gackowski@cm.umk.pl (M.G.)
[3] Doctoral School of Medical and Health Sciences, Ludwik Rydygier Collegium Medicum, Nicolaus Copernicus University in Toruń, Jagiellońska 13-15 Str., 85-067 Bydgoszcz, Poland
[4] Department of Pharmaceutical Chemistry and Biomaterials, Faculty of Pharmacy, Medical University of Warsaw, 1 Banacha Str., 02-093 Warsaw, Poland; mpisklak@wum.edu.pl
[5] Department of Organic and Physical Chemistry, Medical University of Warsaw, 1 Banacha Str., 02-097 Warsaw, Poland
* Correspondence: lukasz.szeleszczuk@wum.edu.pl

Table S1. RMSD and ligand radius of gyration over nominal 50–100 ns for all nineteen runs.

Compound	Run	Backbone RMSD (Å)	Ligand RMSD protein-aligned (Å)	Ligand RMSD self-aligned (Å)	Ligand rGyr (Å)
Apixaban	R1	1.38 ± 0.10	0.90 ± 0.20	0.43 ± 0.10	5.09 ± 0.04
6y	R1	1.35 ± 0.10	4.65 ± 0.23	1.56 ± 0.12	5.11 ± 0.08
7f	R1	1.23 ± 0.09	6.07 ± 0.42	1.99 ± 0.42	5.36 ± 0.20
PAX-M02	R1	1.49 ± 0.12	6.07 ± 0.21	1.32 ± 0.09	5.27 ± 0.08
PAX-M04	R1	1.46 ± 0.15	2.11 ± 0.39	0.83 ± 0.12	4.98 ± 0.10
PAX-M08	R1	1.46 ± 0.10	2.72 ± 0.49	1.34 ± 0.14	5.20 ± 0.10
PAX-M09	R1	1.39 ± 0.11	2.18 ± 0.39	0.82 ± 0.10	5.26 ± 0.08
PAX-M09	R2	1.48 ± 0.12	5.79 ± 0.20	1.42 ± 0.11	5.49 ± 0.07
PAX-M09	R3	1.30 ± 0.10	2.65 ± 0.38	0.85 ± 0.09	5.25 ± 0.07
PAX-M12	R1	1.27 ± 0.12	2.91 ± 1.07	1.24 ± 0.36	5.04 ± 0.18
PAX-M14	R1	1.40 ± 0.12	5.46 ± 0.22	1.38 ± 0.12	5.37 ± 0.06
PAX-M15	R1	1.37 ± 0.10	1.59 ± 0.33	1.20 ± 0.11	5.11 ± 0.07
PAX-M15	R2	1.35 ± 0.11	2.11 ± 0.50	1.27 ± 0.36	5.17 ± 0.14
PAX-M15	R3	1.30 ± 0.09	1.78 ± 0.43	1.02 ± 0.29	5.21 ± 0.10
PAX-M17	R1	1.36 ± 0.13	4.80 ± 0.45	1.37 ± 0.12	4.83 ± 0.07

PAX-M18	R1	1.19 ± 0.09	2.38 ± 0.28	1.27 ± 0.12	5.25 ± 0.09
PAX-M18	R2	1.27 ± 0.09	3.50 ± 0.55	1.61 ± 0.29	5.55 ± 0.18
PAX-M18	R3	1.60 ± 0.10	3.09 ± 0.56	1.23 ± 0.17	5.21 ± 0.07
PAX-M19	R1	1.60 ± 0.13	5.94 ± 0.63	1.54 ± 0.32	5.53 ± 0.17

Table S2. Cα RMSF (Å) for eight specified chain-A residues, calculated over the full exported trajectory.

Compound	Run	Tyr99	Phe174	Asp189	Gln192	Ser195	Trp215	Gly216	Gly218
Apixaban	R1	0.52	0.75	0.78	0.56	0.40	0.36	0.49	0.67
6y	R1	1.37	1.38	0.48	0.51	0.42	0.40	0.48	0.62
7f	R1	0.90	1.44	0.47	0.60	0.47	0.44	0.59	0.70
PAX-M02	R1	0.74	1.06	0.67	0.51	0.43	0.39	0.53	0.79
PAX-M04	R1	0.65	1.73	0.43	0.56	0.37	0.43	0.54	0.81
PAX-M08	R1	0.78	1.85	0.42	0.50	0.41	0.57	0.69	0.87
PAX-M09	R1	0.62	1.23	0.49	0.65	0.51	0.45	0.62	0.77
PAX-M09	R2	0.86	1.49	0.59	0.56	0.42	0.42	0.51	0.85
PAX-M09	R3	0.65	0.90	0.52	0.56	0.38	0.40	0.47	0.70
PAX-M12	R1	0.61	0.75	0.46	0.61	0.46	0.42	0.55	0.93
PAX-M14	R1	1.14	1.61	0.54	0.63	0.37	0.39	0.56	0.86
PAX-M15	R1	0.61	1.07	0.57	0.49	0.39	0.38	0.46	0.84
PAX-M15	R2	0.90	1.34	0.54	0.56	0.46	0.39	0.49	0.74
PAX-M15	R3	0.63	1.10	0.51	0.61	0.46	0.41	0.59	1.05
PAX-M17	R1	0.93	1.09	0.52	0.85	0.48	0.40	0.57	0.76
PAX-M18	R1	1.01	1.90	0.44	0.56	0.43	0.42	0.54	0.76
PAX-M18	R2	0.52	1.07	0.42	0.49	0.36	0.38	0.47	0.65
PAX-M18	R3	1.13	2.70	0.57	0.72	0.45	0.55	0.60	0.83
PAX-M19	R1	1.35	1.89	0.47	0.59	0.51	0.58	0.63	0.67

Table S3. Selected binary residue occupancies (%) over nominal 50–100 ns.

Compound	Run	Gly216 H-bond	Gly218 H-bond	Ser195 water bridge	Trp215 π–π	Trp215 hydrophobic union
Apixaban	R1	93.4	0.0	0.0	0.4	56.1
6y	R1	99.6	98.4	95.4	0.0	3.6
7f	R1	2.6	60.3	0.0	63.9	80.8

PAX-M02	R1	97.6	94.2	83.2	0.0	8.0
PAX-M04	R1	10.8	65.1	5.4	69.7	87.2
PAX-M08	R1	21.4	0.0	8.4	45.7	82.6
PAX-M09	R1	7.2	3.0	30.9	93.4	97.0
PAX-M09	R2	86.8	89.8	80.6	0.0	9.2
PAX-M09	R3	1.6	1.0	16.2	98.4	99.8
PAX-M12	R1	14.2	1.2	17.4	49.7	79.8
PAX-M14	R1	98.8	92.4	80.2	0.0	10.4
PAX-M15	R1	38.3	64.7	70.9	80.2	93.6
PAX-M15	R2	51.9	49.1	74.5	55.7	75.6
PAX-M15	R3	48.9	51.9	71.3	44.5	74.9
PAX-M17	R1	0.0	0.0	0.0	90.4	96.6
PAX-M18	R1	30.9	25.1	67.5	53.9	83.0
PAX-M18	R2	62.1	80.6	81.6	8.6	40.1
PAX-M18	R3	10.0	16.4	32.7	62.7	87.4
PAX-M19	R1	41.1	3.2	69.3	24.2	76.6

Table S4. Contact presence over nominal 80–100 ns (201 saved frames).

Compound	Run	Any protein contact (%)	Site contact including water (%)	Direct site contact (%)	Max. consecutive site-negative frames
Apixaban	R1	100.0	100.0	100.0	0
6y	R1	100.0	100.0	100.0	0
7f	R1	99.5	99.0	98.5	1
PAX-M02	R1	100.0	100.0	100.0	0
PAX-M04	R1	99.5	99.5	97.0	1
PAX-M08	R1	100.0	94.0	92.5	1
PAX-M09	R1	100.0	100.0	100.0	0
PAX-M09	R2	100.0	99.0	97.5	1
PAX-M09	R3	100.0	100.0	100.0	0
PAX-M12	R1	100.0	100.0	99.5	0
PAX-M14	R1	100.0	100.0	100.0	0
PAX-M15	R1	100.0	100.0	99.5	0
PAX-M15	R2	100.0	100.0	99.0	0
PAX-M15	R3	100.0	100.0	99.0	0

Compound	Run	Any protein contact (%)	Site contact including water (%)	Direct site contact (%)	Max. consecutive site-negative frames
PAX-M17	R1	100.0	100.0	100.0	0
PAX-M18	R1	100.0	100.0	99.5	0
PAX-M18	R2	100.0	100.0	100.0	0
PAX-M18	R3	100.0	99.5	99.0	1
PAX-M19	R1	100.0	95.5	90.0	3

A. Apixaban — run 1

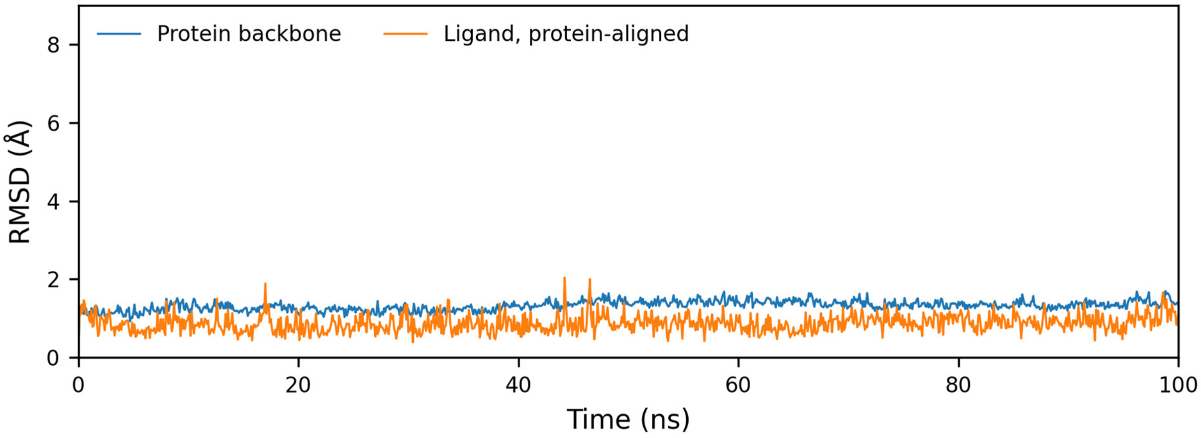

B. 6y — run 1

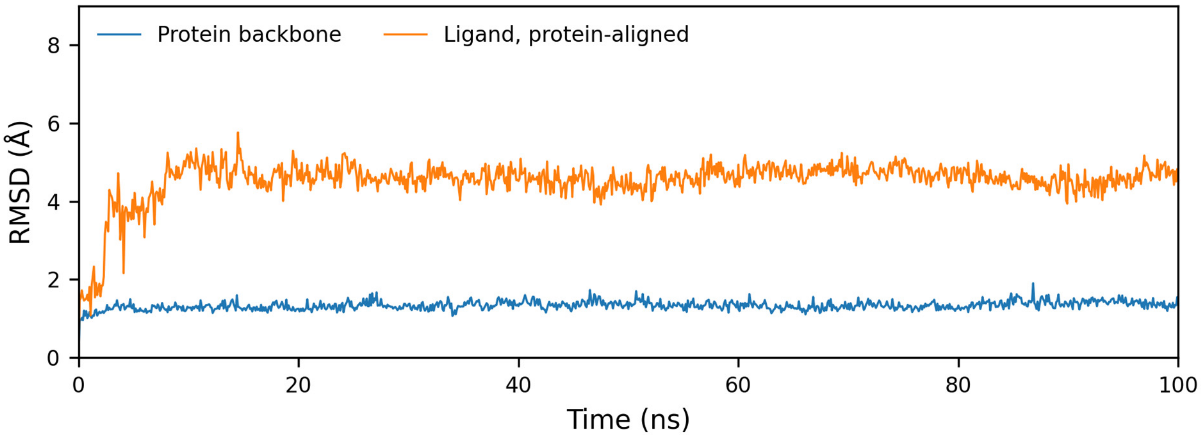

C. 7f — run 1

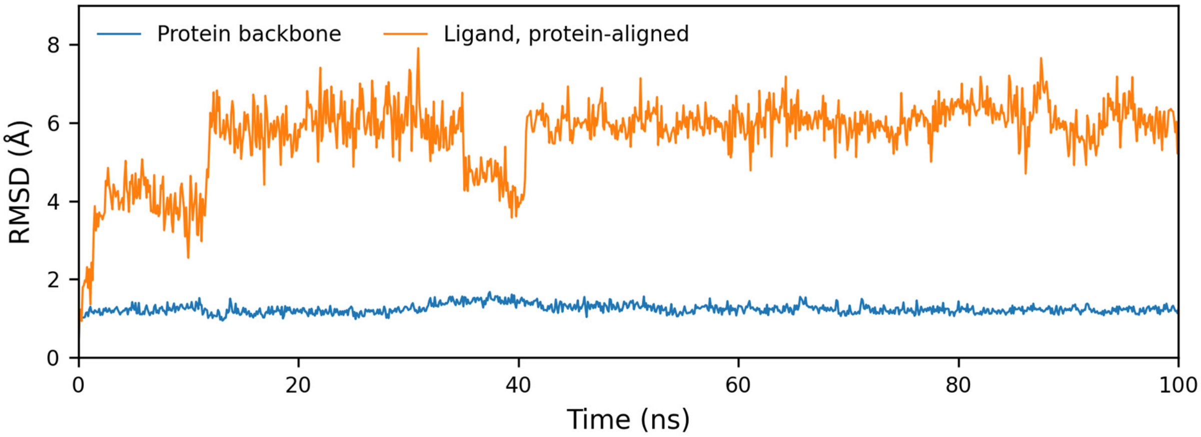

D. PAX-M02 — run 1

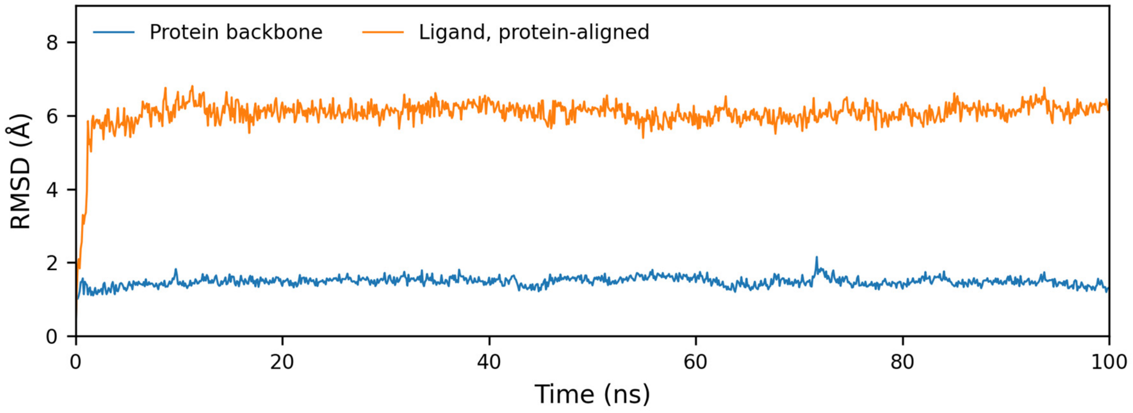

E. PAX-M04 — run 1

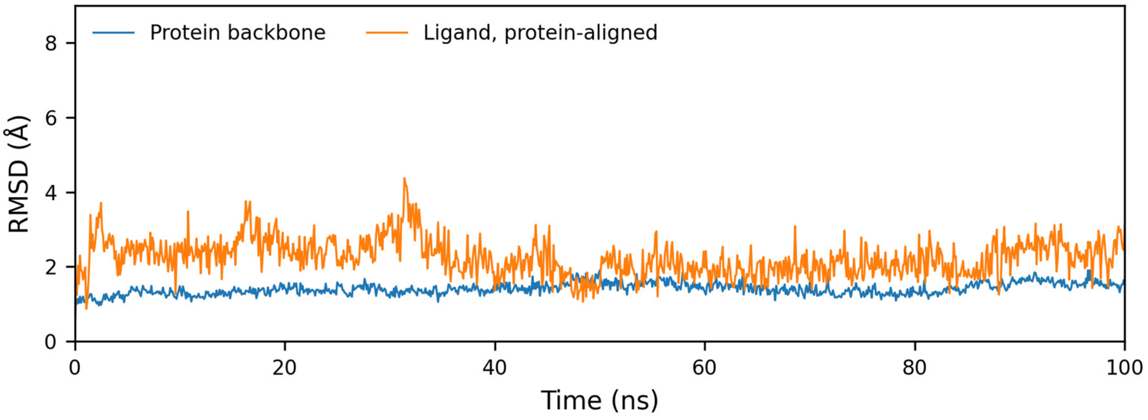

F. PAX-M08 — run 1

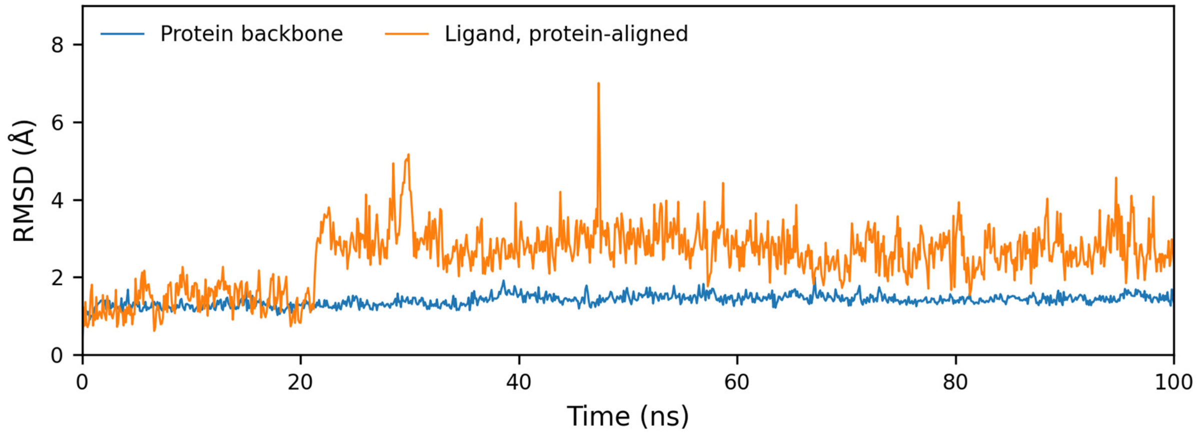

G. PAX-M09 — run 1

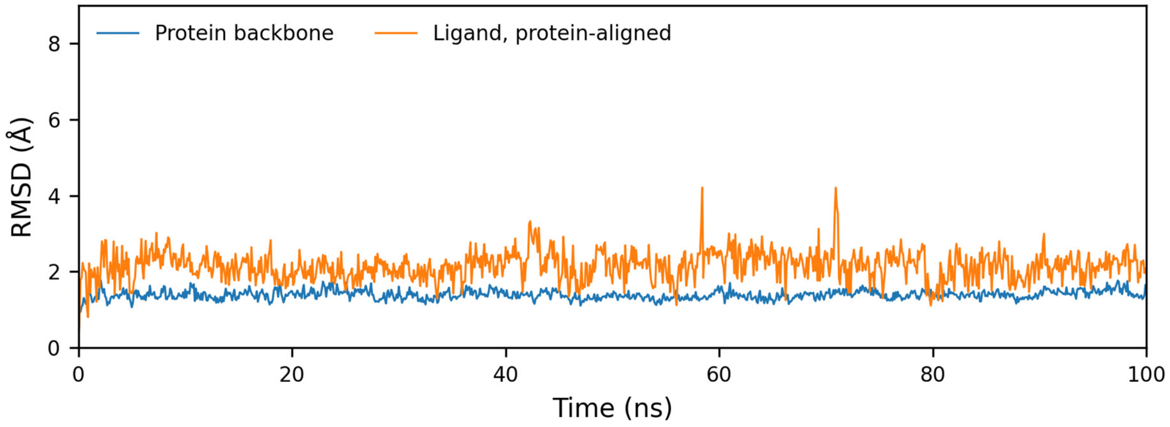

H. PAX-M12 — run 1

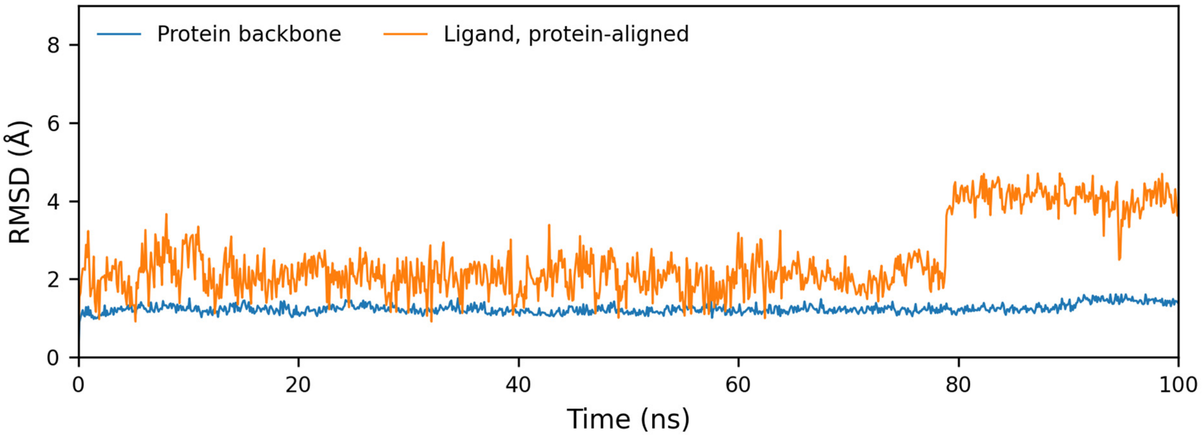

I. PAX-M14 — run 1

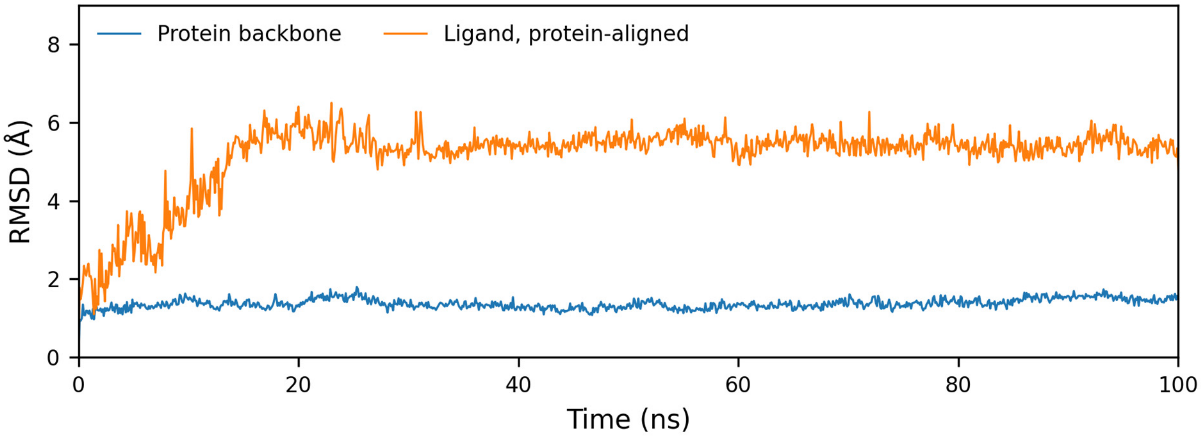

J. PAX-M15 — run 1

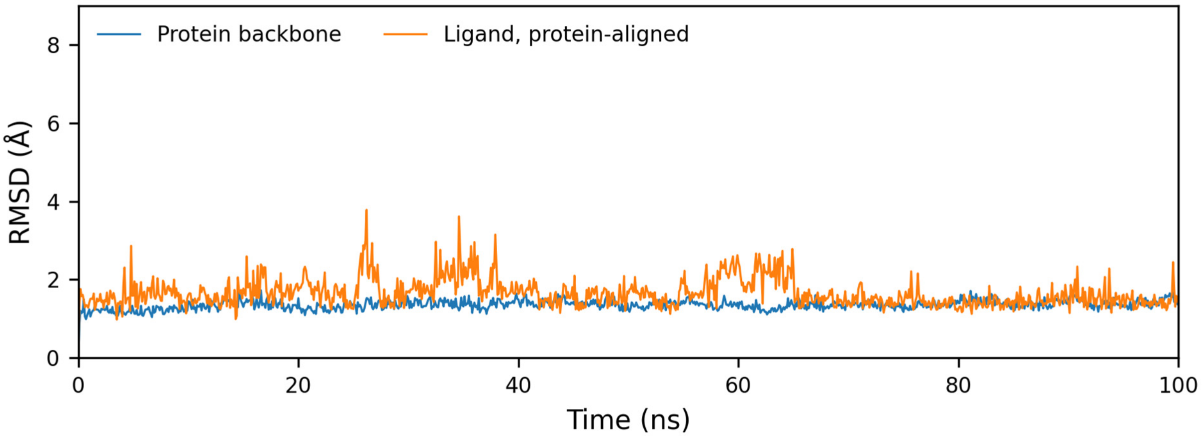

K. PAX-M17 — run 1

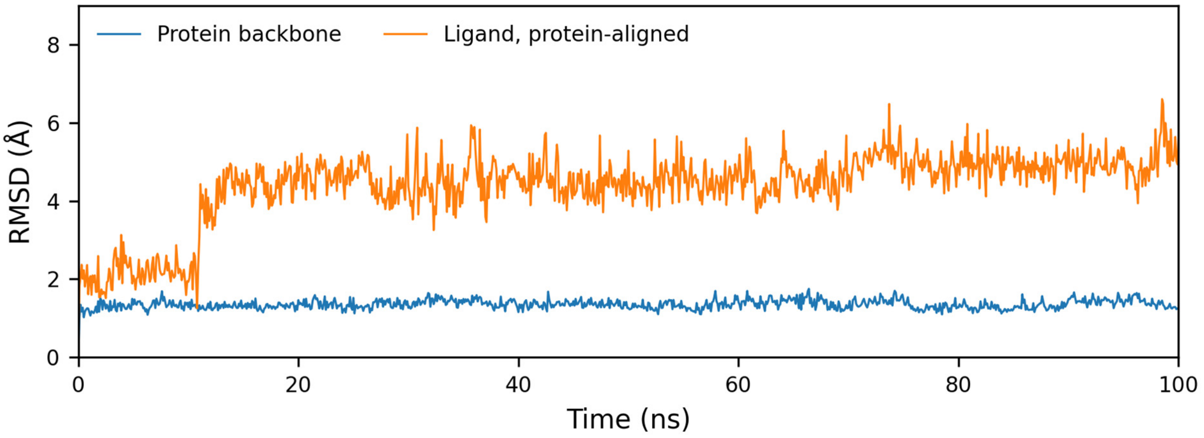

L. PAX-M18 — run 1

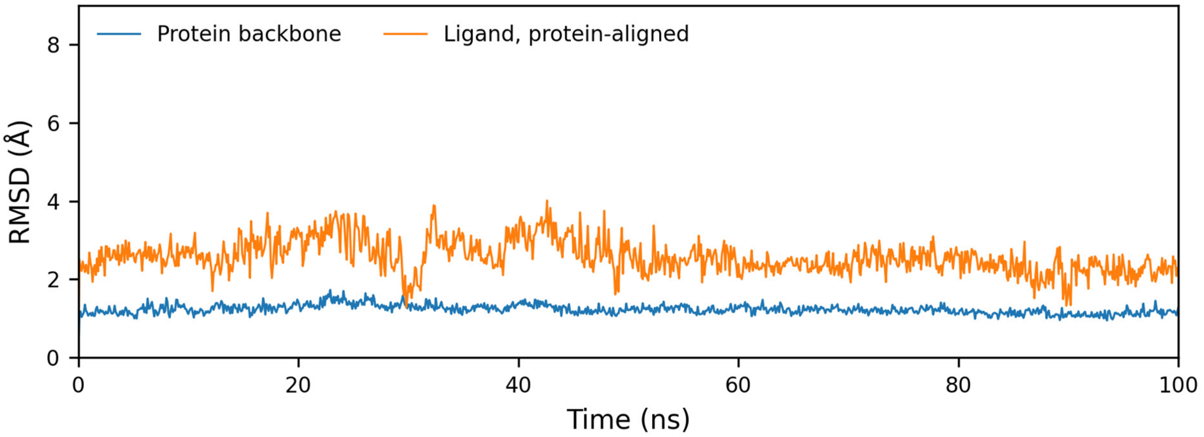

M. PAX-M19 — run 1

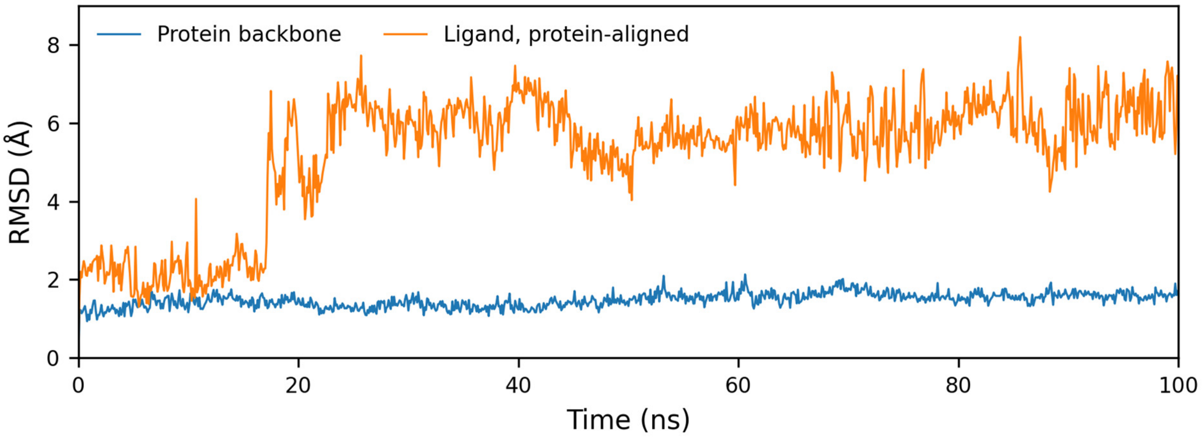

Figure S1. Protein-backbone and protein-aligned ligand RMSD profiles for the thirteen initial nominal 100 ns simulations. The two quantities share the same Å axis; ligand self-aligned RMSD is reported separately in Table S1. Panels: (A) Apixaban, R1; (B) 6y, R1; (C) 7f, R1; (D) PAX-M02, R1; (E) PAX-M04, R1; (F) PAX-M08, R1; (G) PAX-M09, R1; (H) PAX-M12, R1; (I) PAX-M14, R1; (J) PAX-M15, R1; (K) PAX-M17, R1; (L) PAX-M18, R1; (M) PAX-M19, R1.

A. Apixaban — run 1

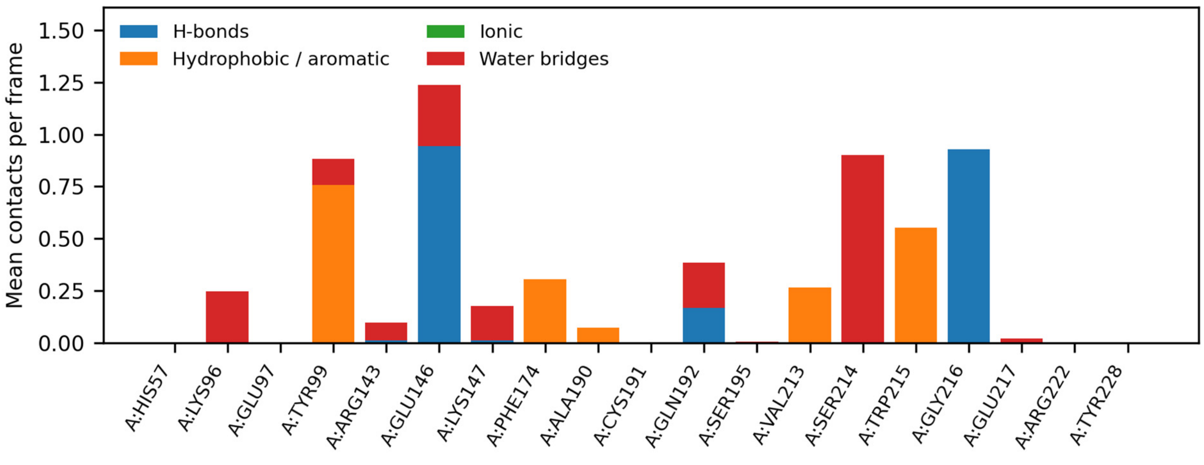

B. 6y — run 1

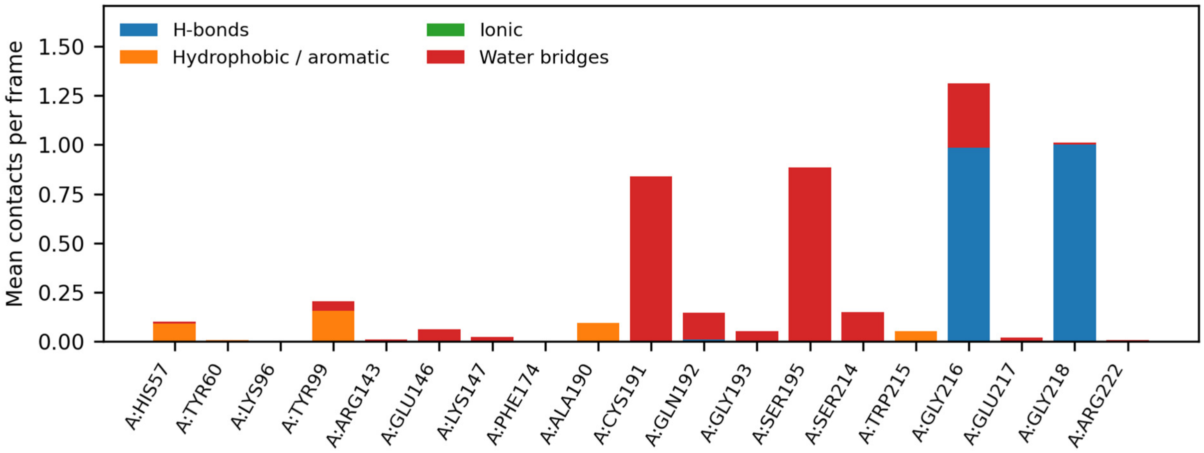

C. 7f — run 1

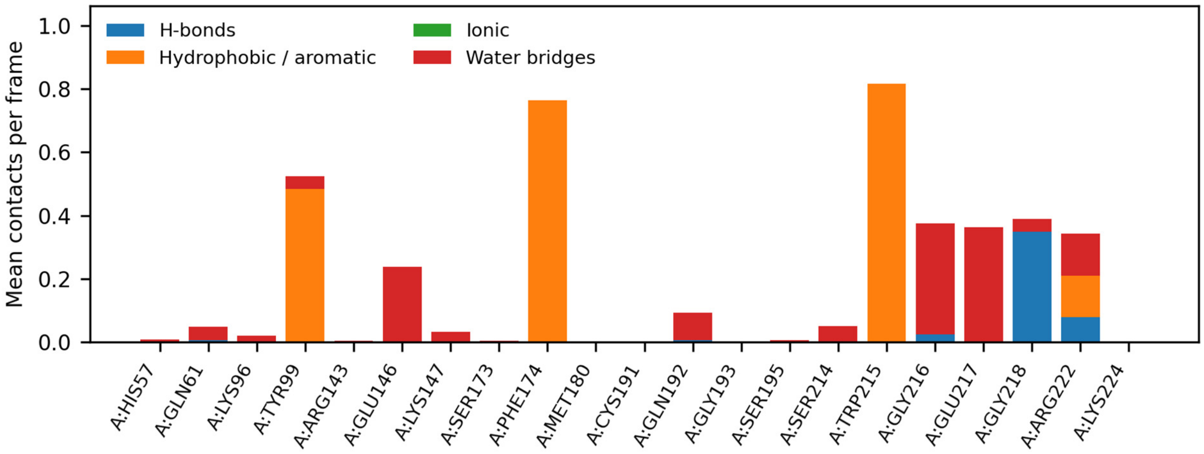

D. PAX-M02 — run 1

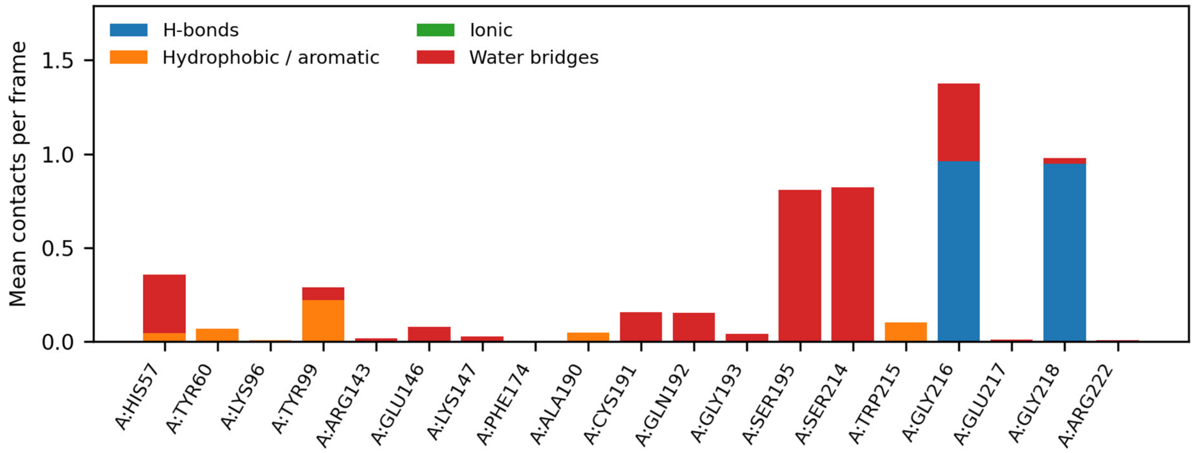

E. PAX-M04 — run 1

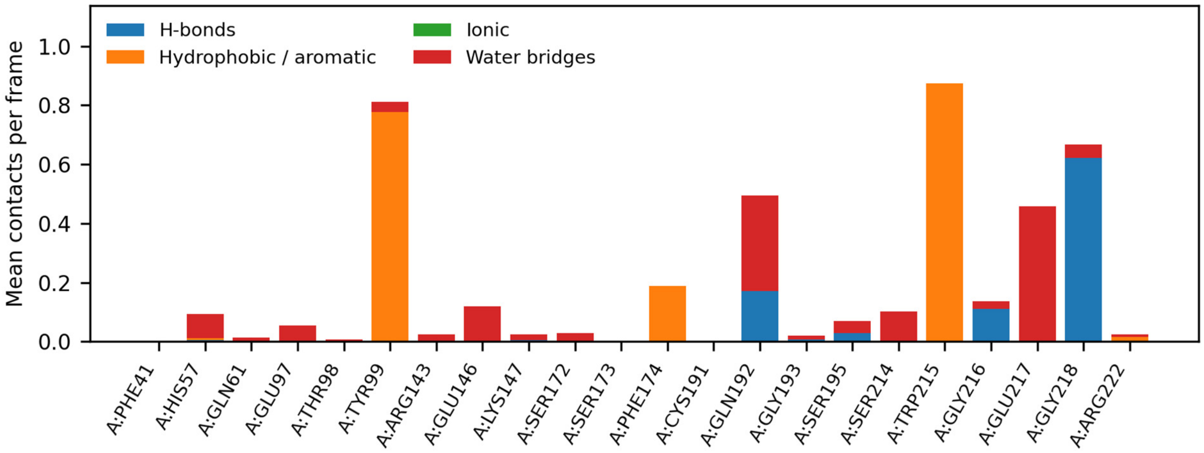

F. PAX-M08 — run 1

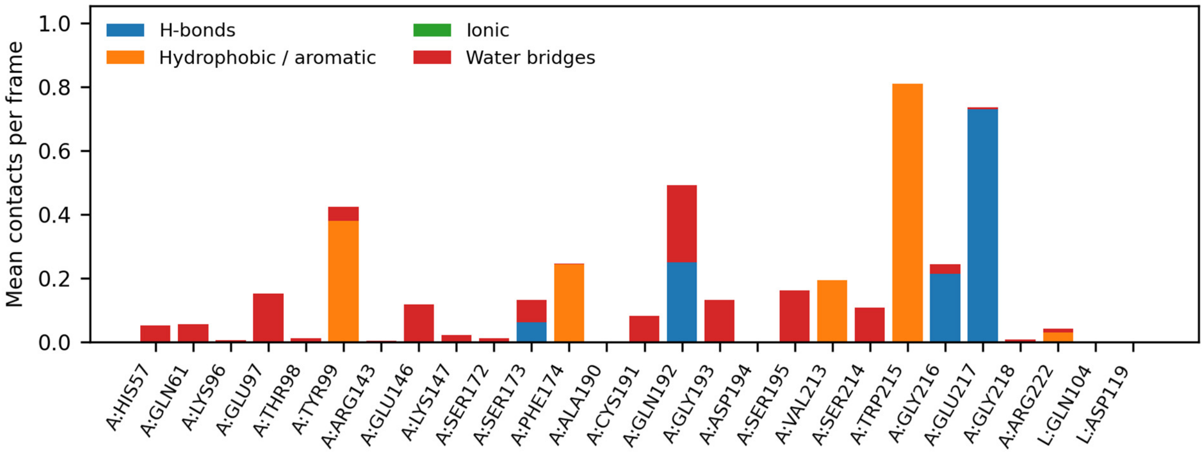

G. PAX-M09 — run 1

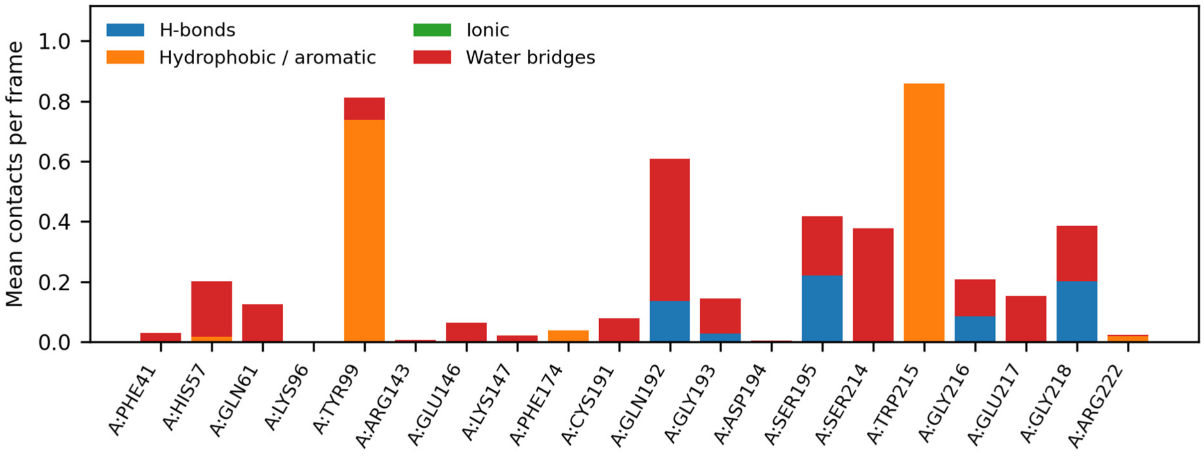

H. PAX-M12 — run 1

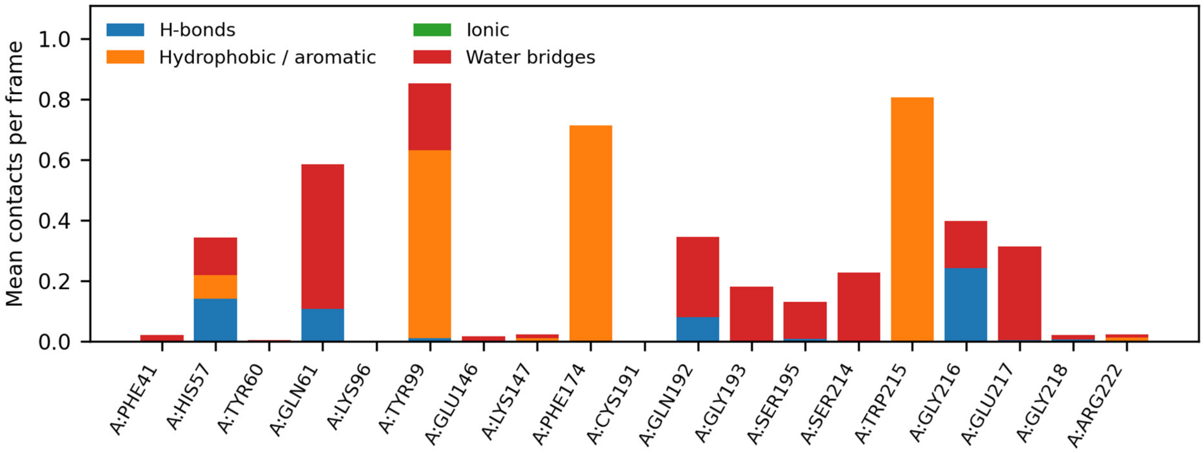

I. PAX-M14 — run 1

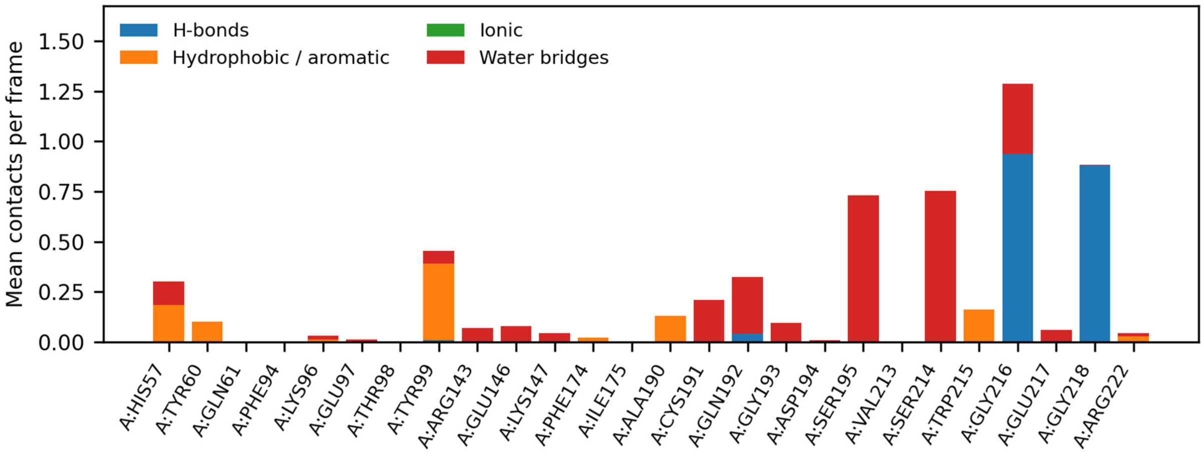

J. PAX-M15 — run 1

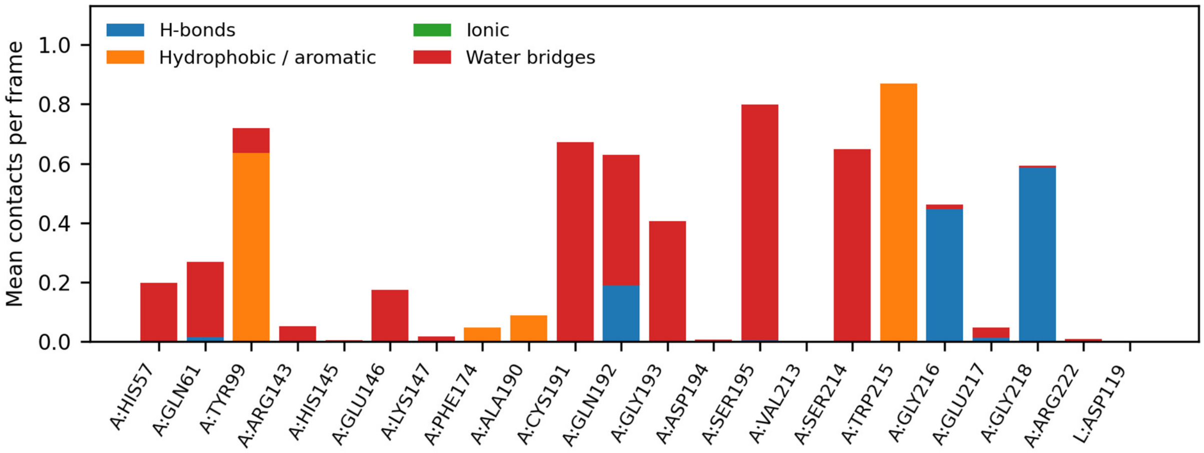

K. PAX-M17 — run 1

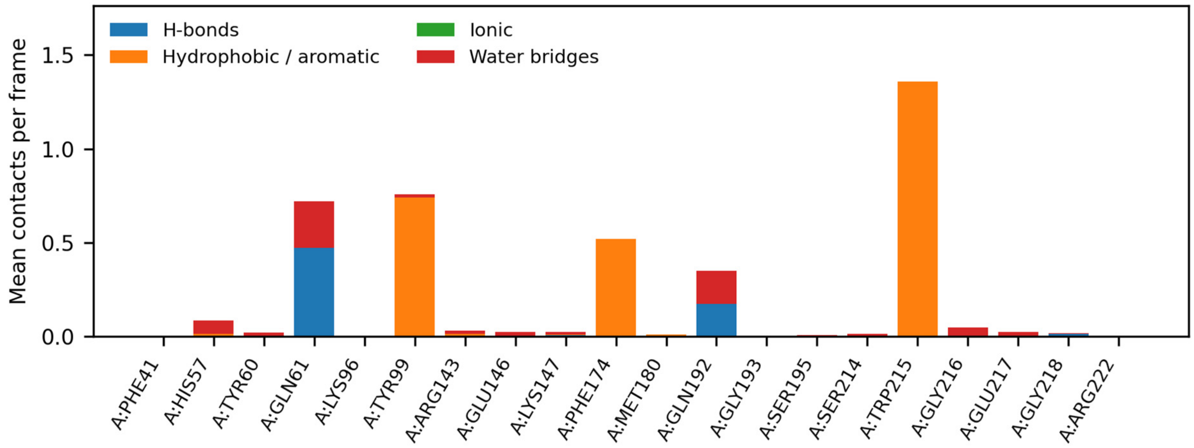

L. PAX-M18 — run 1

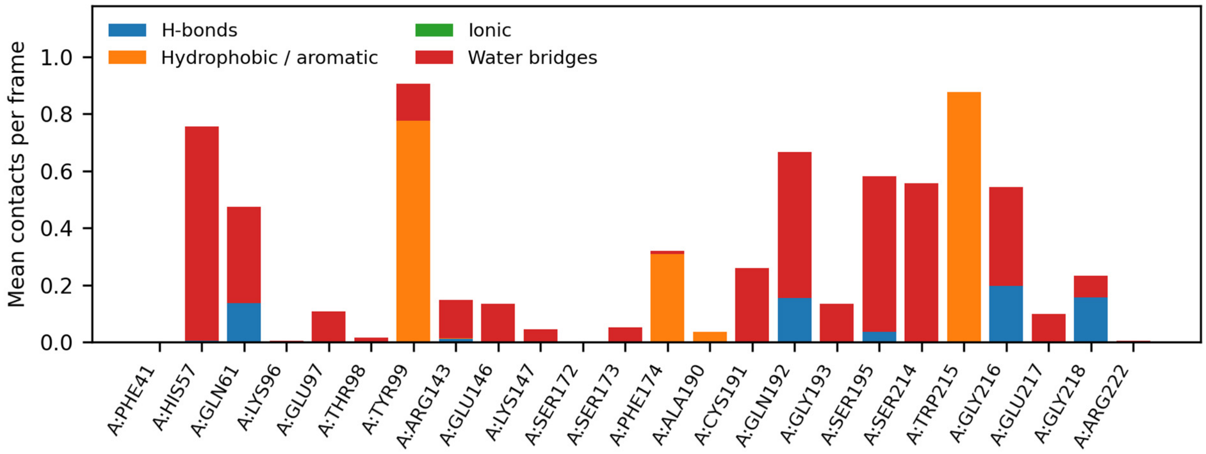

M. PAX-M19 — run 1

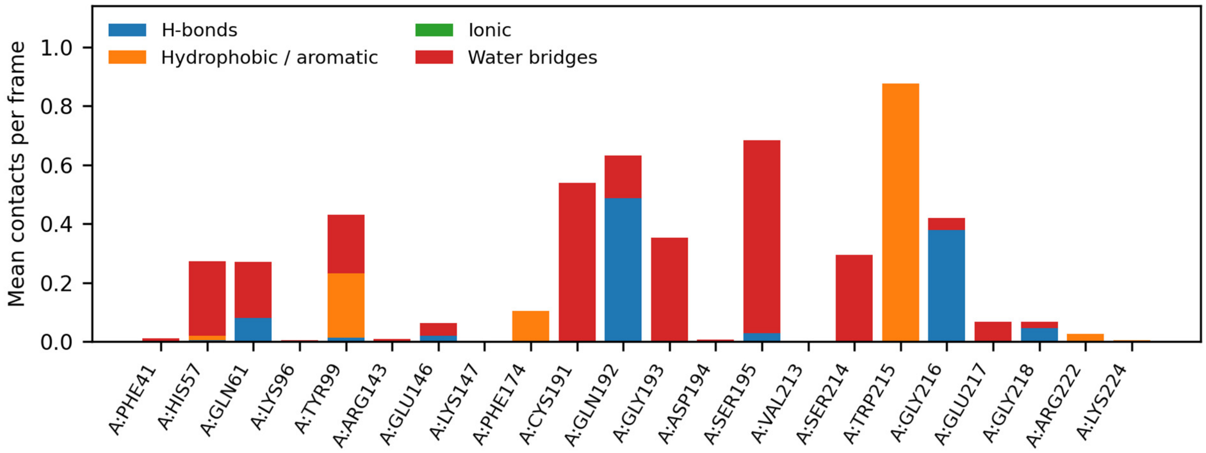

Figure S2. Full-trajectory protein–ligand contact-count distributions for the initial runs. Stacks show hydrogen bonds, hydrophobic/aromatic interactions, ionic contacts, and water bridges. Heights are mean counts per frame, not binary probabilities; concurrent contacts can produce values above one. All exported residue categories are retained. Panels: (A) Apixaban, R1; (B) 6y, R1; (C) 7f, R1; (D) PAX-M02, R1; (E) PAX-M04, R1; (F) PAX-M08, R1; (G) PAX-M09, R1; (H) PAX-M12, R1; (I) PAX-M14, R1; (J) PAX-M15, R1; (K) PAX-M17, R1; (L) PAX-M18, R1; (M) PAX-M19, R1.

A. PAX-M09 — run 1

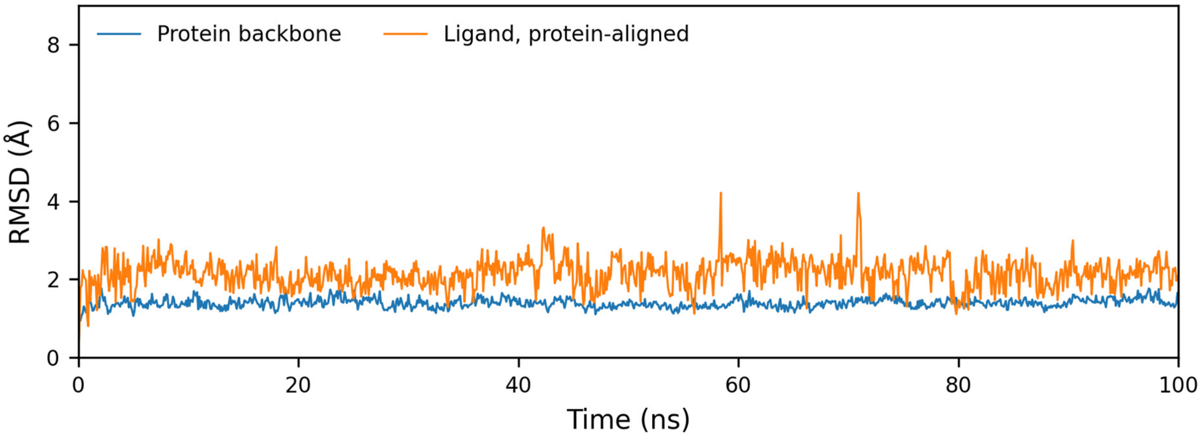

B. PAX-M09 — run 2

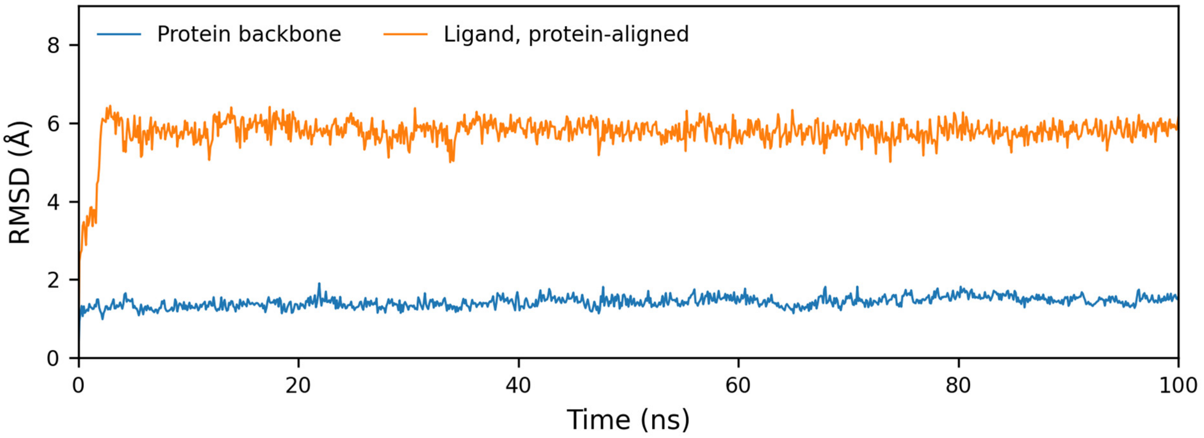

C. PAX-M09 — run 3

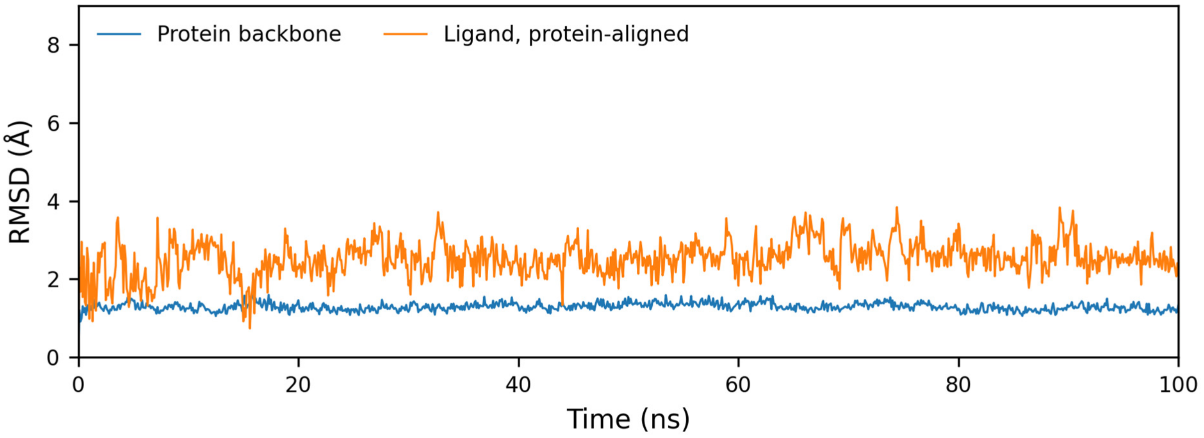

D. PAX-M15 — run 1

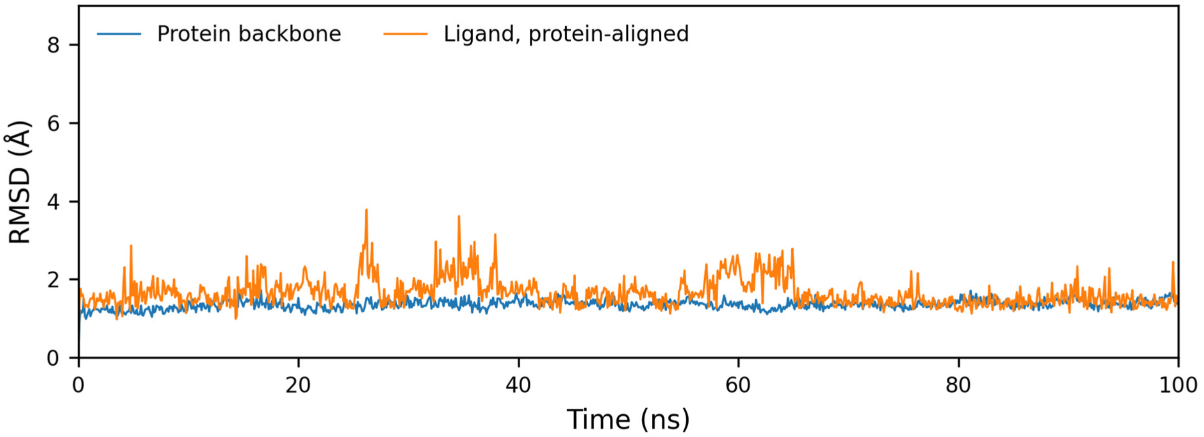

E. PAX-M15 — run 2

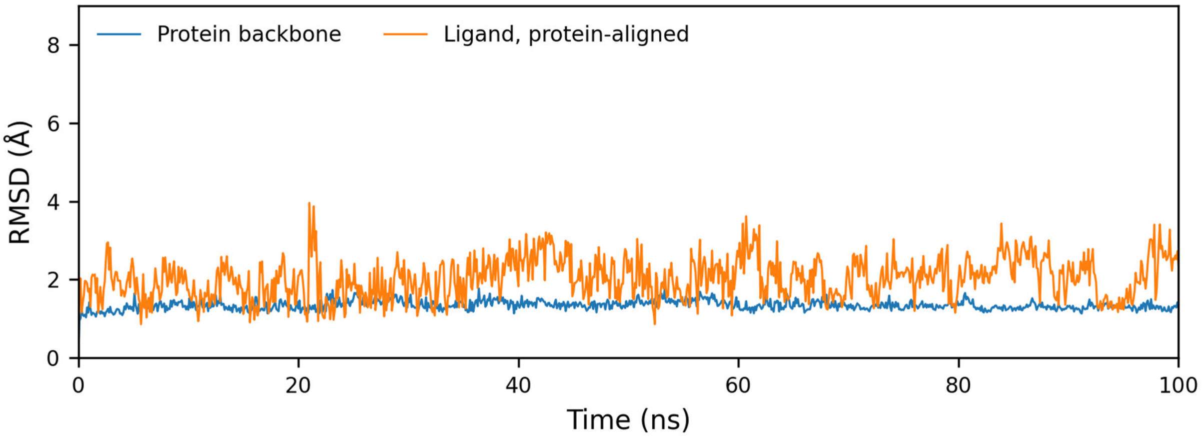

F. PAX-M15 — run 3

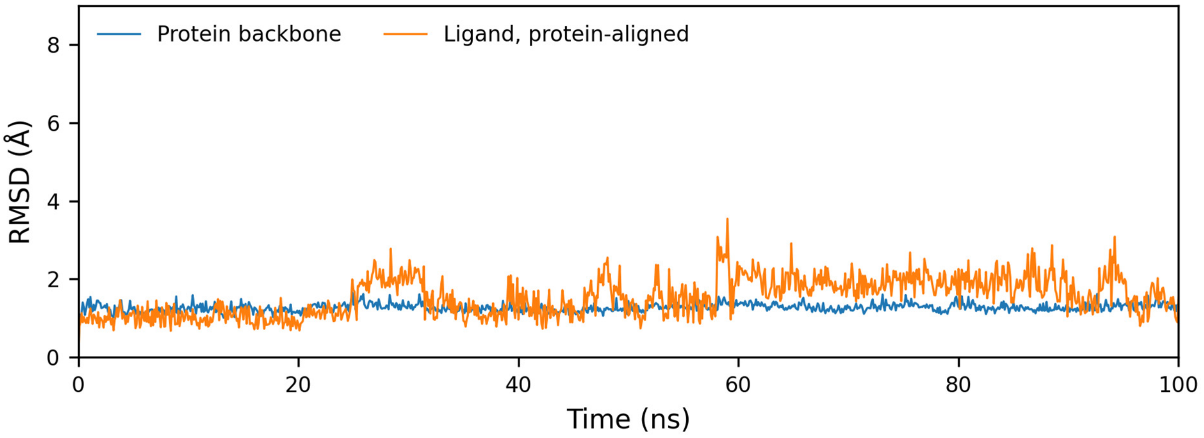

G. PAX-M18 — run 1

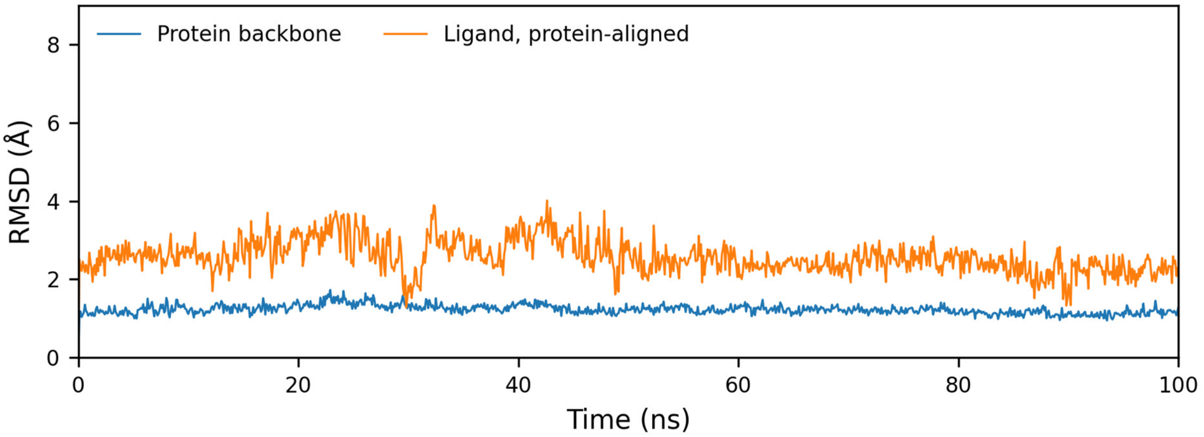

H. PAX-M18 — run 2

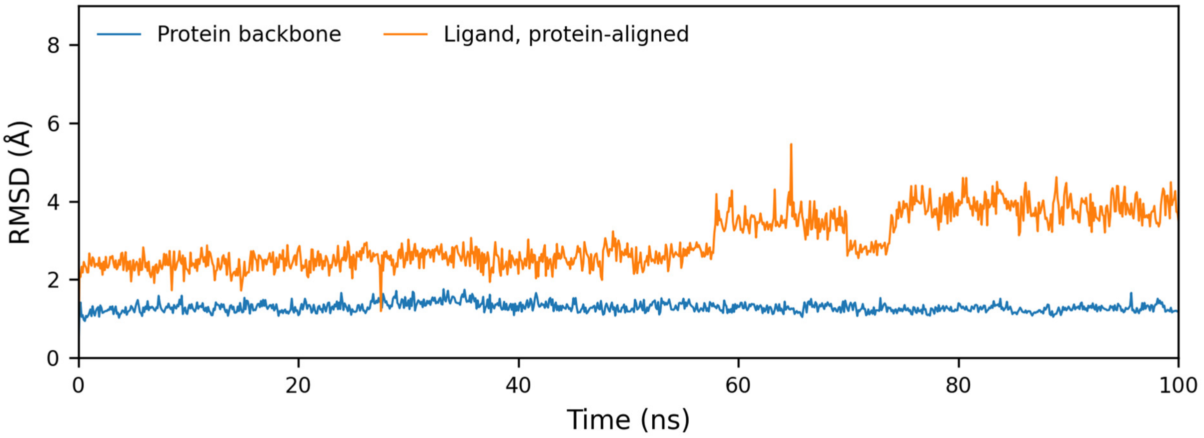

I. PAX-M18 — run 3

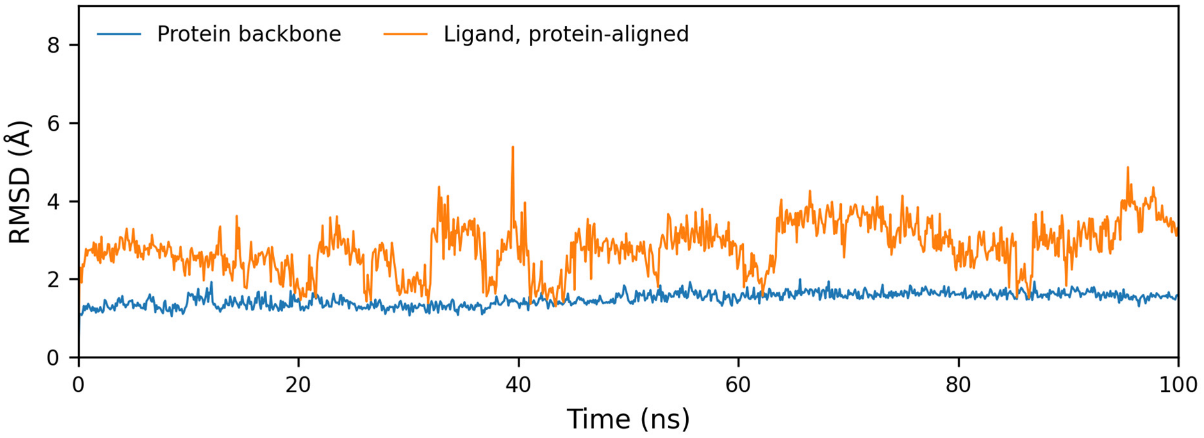

Figure S3. Protein-backbone and protein-aligned ligand RMSD for the three independent nominal 100 ns runs of each selected PAX compound. Initial runs are repeated from Figure S1 to facilitate within-compound comparison. Panels: (A) PAX-M09, R1; (B) PAX-M09, R2; (C) PAX-M09, R3. (D) PAX-M15, R1; (E) PAX-M15, R2; (F) PAX-M15, R3; (G) PAX-M18, R1; (H) PAX-M18, R2; (I) PAX-M18, R3.

A. PAX-M09 — run 1

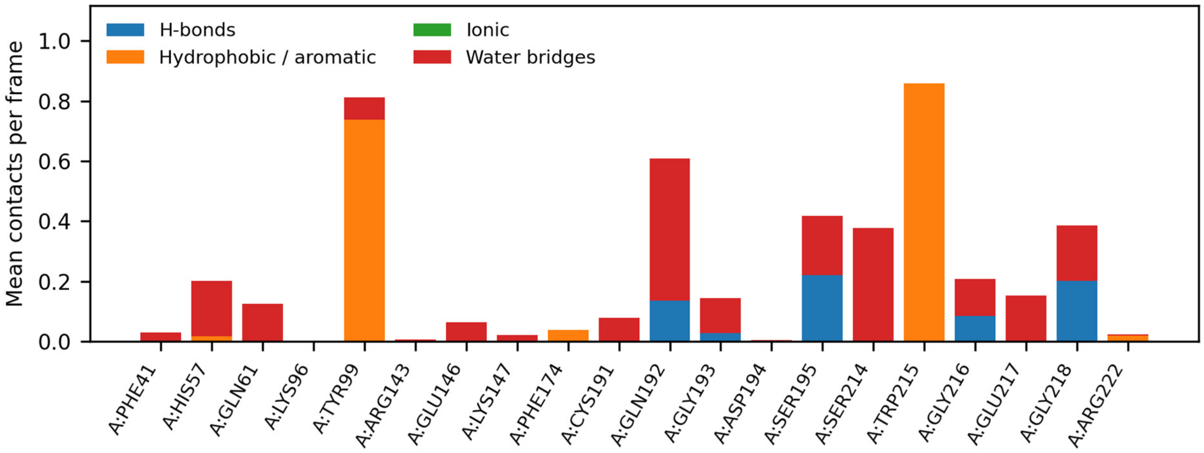

B. PAX-M09 — run 2

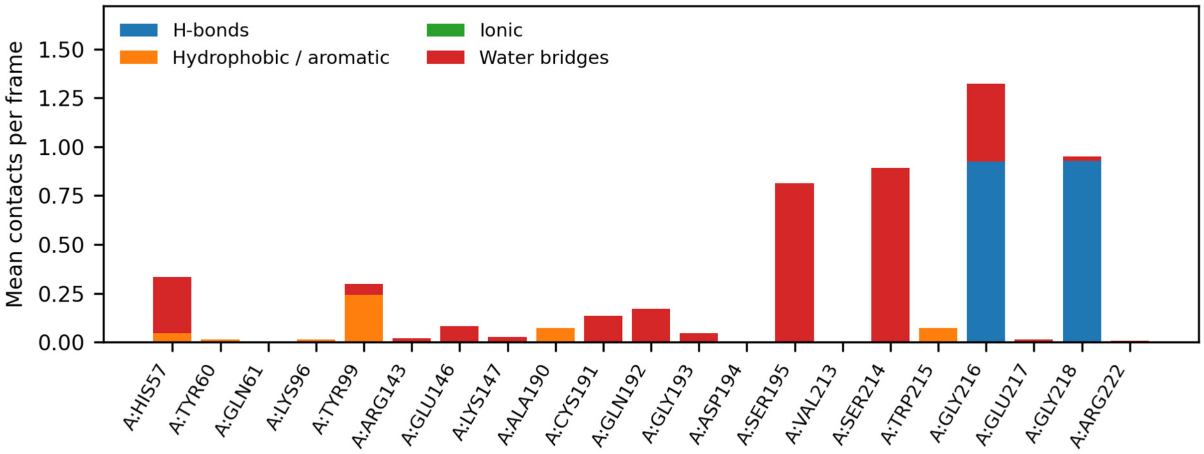

C. PAX-M09 — run 3

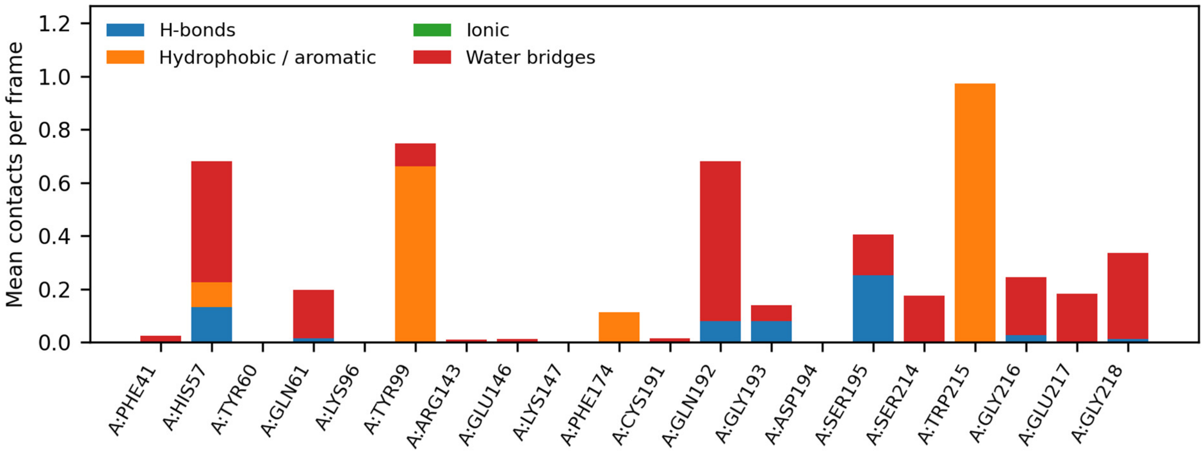

D. PAX-M15 — run 1

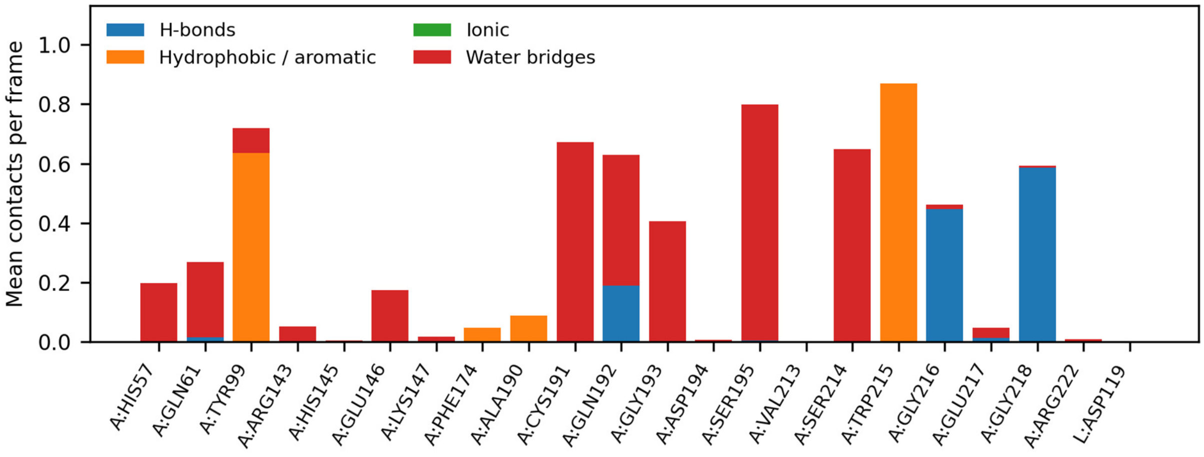

E. PAX-M15 — run 2

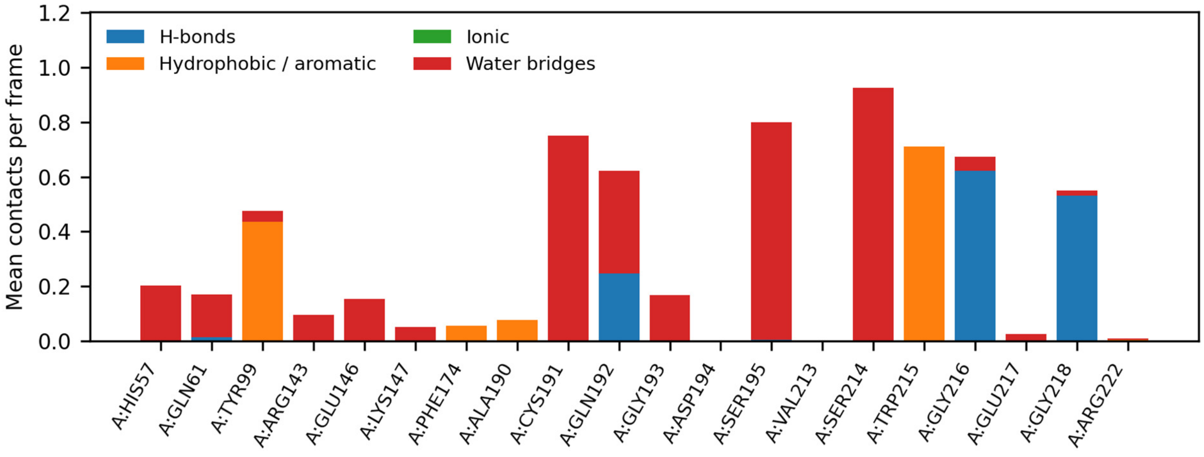

F. PAX-M15 — run 3

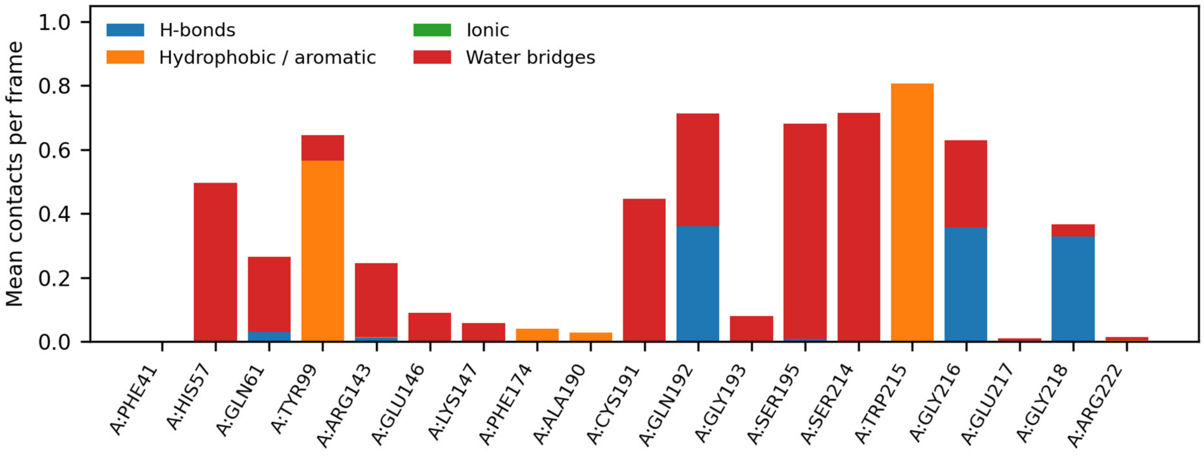

G. PAX-M18 — run 1

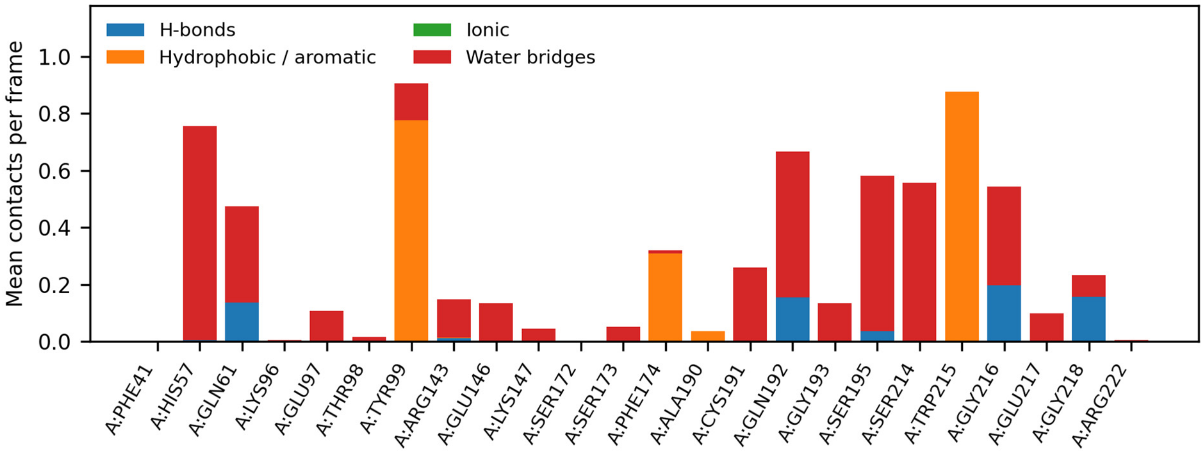

H. PAX-M18 — run 2

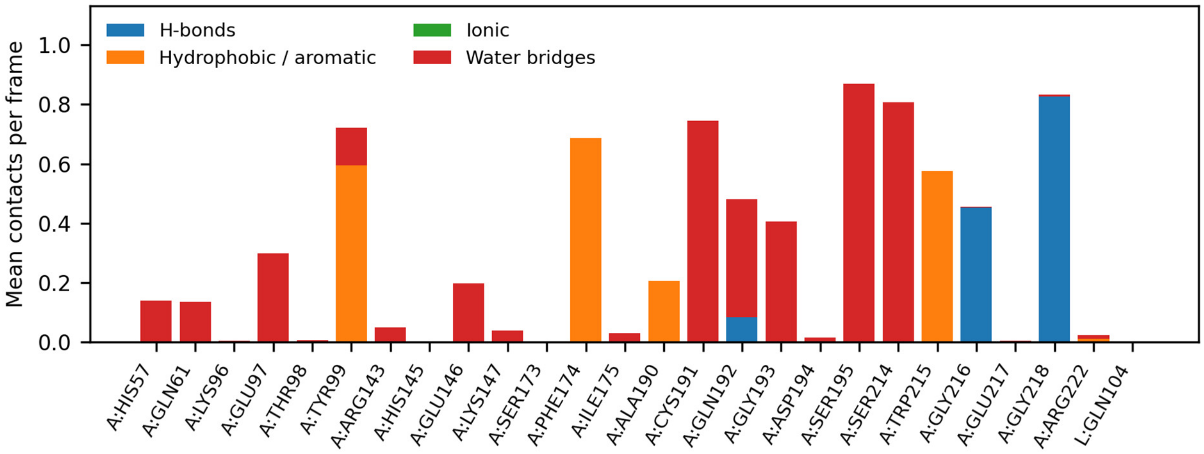

I. PAX-M18 — run 3

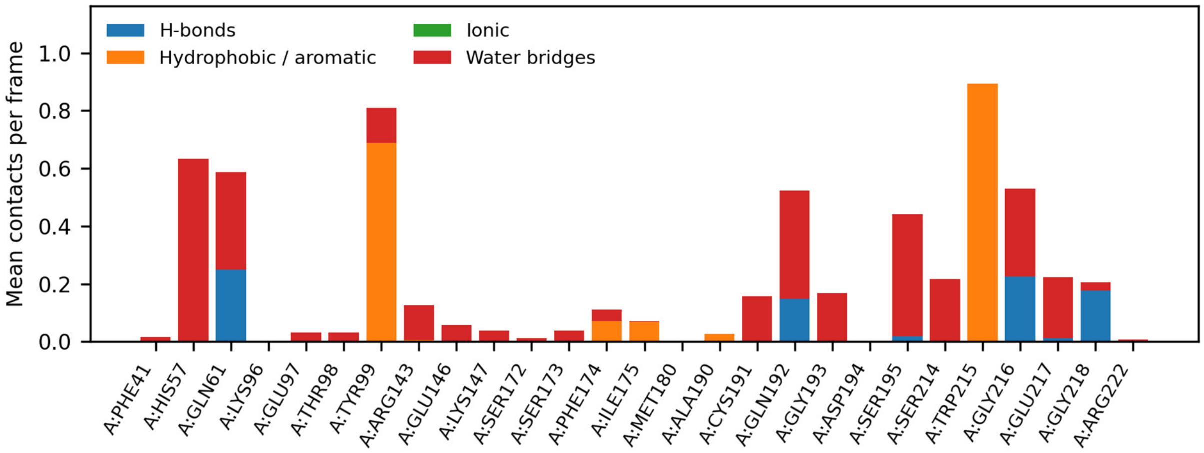

Figure S4. Full-trajectory contact-count distributions for the three runs of each selected PAX compound. Initial-run histograms are repeated from Figure S2 for direct comparison. Category definitions match Figure S2; window-specific binary occupancies are reported separately in Table S3. Panels: (A) PAX-M09, R1; (B) PAX-M09, R2; (C) PAX-M09, R3; (D) PAX-M15, R1; (E) PAX-M15, R2; (F) PAX-M15, R3; (G) PAX-M18, R1; (H) PAX-M18, R2; (I) PAX-M18, R3.
